# Correlation analysis of disulfidptosis-related gene signatures with clinical prognosis and immunotherapy response in sarcoma

**DOI:** 10.1038/s41598-024-57594-x

**Published:** 2024-03-26

**Authors:** Juan Xu, Kangwen Guo, Xiaoan Sheng, Yuting Huang, Xuewei Wang, Juanjuan Dong, Haotian Qin, Chao Wang

**Affiliations:** 1 Department of Oncology, Chaohu Hospital of Anhui Medical University, Hefei, China; 2 Affiliated Hospital of Guangdong Medical University, Zhanjiang, China; 3 National and Local Joint Engineering Research Center of Orthopaedic Biomaterials, Peking University Shenzhen Hospital, Shenzhen, China; 4 Department of Bone and Joint Surgery, Peking University Shenzhen Hospital, Shenzhen, China

**Keywords:** Disulfidptosis, Riskscore model, Prognosis, Immunotherapy response, Sarcoma, Cancer, Cell biology, Drug discovery, Genetics, Immunology, Molecular biology, Oncology, Risk factors

## Abstract

Disulfidptosis, a newly discovered type of programmed cell death, could be a mechanism of cell death controlled by SLC7A11. This could be closely associated with tumor development and advancement. Nevertheless, the biological mechanism behind disulfidptosis-related genes (DRGs) in sarcoma (SARC) is uncertain. This study identified three valuable genes (*SLC7A11, RPN1, GYS1*) associated with disulfidptosis in sarcoma (SARC) and developed a prognostic model. The multiple databases and RT-qPCR data confirmed the upregulated expression of prognostic DRGs in SARC. The TCGA internal and ICGC external validation cohorts were utilized to validate the predictive model capacity. Our analysis of DRG riskscores revealed that the low-risk group exhibited a more favorable prognosis than the high-risk group. Furthermore, we observed a significant association between DRG riskscores and different clinical features, immune cell infiltration, immune therapeutic sensitivity, drug sensitivity, and RNA modification regulators. In addition, two external independent immunetherapy datasets and clinical tissue samples were collected, validating the value of the DRGs risk model in predicting immunotherapy response. Finally, the SLC7A11/hsa-miR-29c-3p/LINC00511, and RPN1/hsa-miR-143-3p/LINC00511 regulatory axes were constructed. This study provided DRG riskscore signatures to predict prognosis and response to immunotherapy in SARC, guiding personalized treatment decisions.

## Introduction

Sarcoma encompasses various malignant neoplasms arising from stromal or connective tissue. With over 100 distinct subtypes, these tumors can manifest in multiple anatomical sites. Due to its rarity and diagnostic challenges, metastasis often transpires during the underdiagnosed disease stage^1^. However, the survival rate for localized sarcoma can become 70–75% by employing a comprehensive approach associated with systemic therapy, chemotherapy, and effective management of the primary tumor via surgery and/or radiation^2^. Despite receiving standard treatment, most patients succumb to tumor recurrence and metastasis, primarily due to inadequate response to current therapeutic approaches^3^. The persisting challenges of drug resistance, unfavorable prognosis, and other unresolved issues necessitate developing novel diagnostic markers with heightened efficiency and sensitivity to evaluate the prognosis of SARC patients. Additionally, innovative and more productive treatment modalities are imperative to enhance their overall prognosis.

Recently, a new type of cell death called disulfidptosis, independent of the known apoptosis, necrosis, and copper-based apoptosis, has been discovered. It was observed that in cells exhibiting elevated levels of SLC7A11 expression during glucose deprivation, NADPH availability was insufficient to support the cellular reduction of cystine to cysteine. Therefore, an abnormal cystine accumulation and other disulfide compounds would ensue inside the cell, leading to disulfide stress. This stress could induce the formation of disulfide bond crosslinks within actin cytoskeletal proteins, causing cytoskeletal contraction and subsequent cell plasma membrane detachment. Ultimately, these events would culminate in cellular demise^4,5^. The SLC7A11 transporter facilitates the translocation of extracellular cystine into cells while maintaining a 1:1 ratio with intracellular glutamate. Extensive evidence has indicated that SLC7A11 overexpression within cancer cells propagates disulfide-induced cell death post-glucose deprivation^6,7^. Moreover, SLC7A11 is pivotal in maintaining cellular redox homeostasis, modulating ferroptosis, and affecting tumor drug resistance across different pathophysiological processes^8^. Recent studies have found a close association between disulfide-mediated cell death and the occurrence and progression of osteosarcoma. Guo et al.^9^ revealed a novel mechanism whereby super-enhancer-driven MLX re-establishes cysteine metabolism in osteosarcoma cells through SLC7A11 to cope with oxidative stress, highlighting the feasibility and clinical prospects of targeting SLC7A11 in osteosarcoma. He et al.^10^ reported that circKIF4A enhances osteosarcoma proliferation and metastasis by sponging miR-515-5p and upregulating SLC7A11. Yue et al.^11^ found that diallyl disulfide inhibits proliferation, apoptosis, invasion, and metastasis of human osteosarcoma cells by modulating the PI3K/Akt/mTOR signaling pathway. Diallyl disulfide upregulates miR-134 to inhibit FOXM1-mediated osteosarcoma proliferation and invasion^12^. Therefore, targeting disulfide-mediated apoptosis may be a potential approach for cancer therapy. However, the pathophysiological connections between disulfide-mediated cell death and apoptosis remain incompletely elucidated. Consequently, disulfide-mediated cell death holds potential as a novel domain for cancer therapy, potentially serving as a new strategy for the clinical treatment of osteosarcoma. However, additional exploration is imperative to decipher its potential mechanisms and clinical applicability.

Recently, innovative immunotherapy-based treatments have depicted noteworthy therapeutic outcomes across diverse solid tumors, prompting various immunotherapeutic approaches for sarcoma treatment^13^. From a clinical perspective, the effectiveness of combined immunotherapy could be seen in specific sarcoma subtypes, such as adenosquamous lung carcinoma, angiosarcoma, and undifferentiated sarcomas^14,15^. Consequently, investigating the mechanisms underlying immune infiltration within sarcoma and determining the appropriate immune checkpoint inhibitors (ICIs) for targeted medications and radiotherapy/chemotherapy could offer novel insights into sarcoma patients.

The current study performed a thorough bioinformatics analysis of DRGs in SARC and employed RT-qPCR for experimental validation. The investigation examined the association between the expression of disulfidptosis-related genes and SARC prognosis and the tumor microenvironment. A novel prognostic signature was developed depending on three DRGs, and a ceRNA regulatory network was constructed. These research findings will provide valuable insights for the therapeutic and prognostic evaluation of SARC.

## Results

### Identification and analysis of DRG clusters in SARC

**Figure 1:**
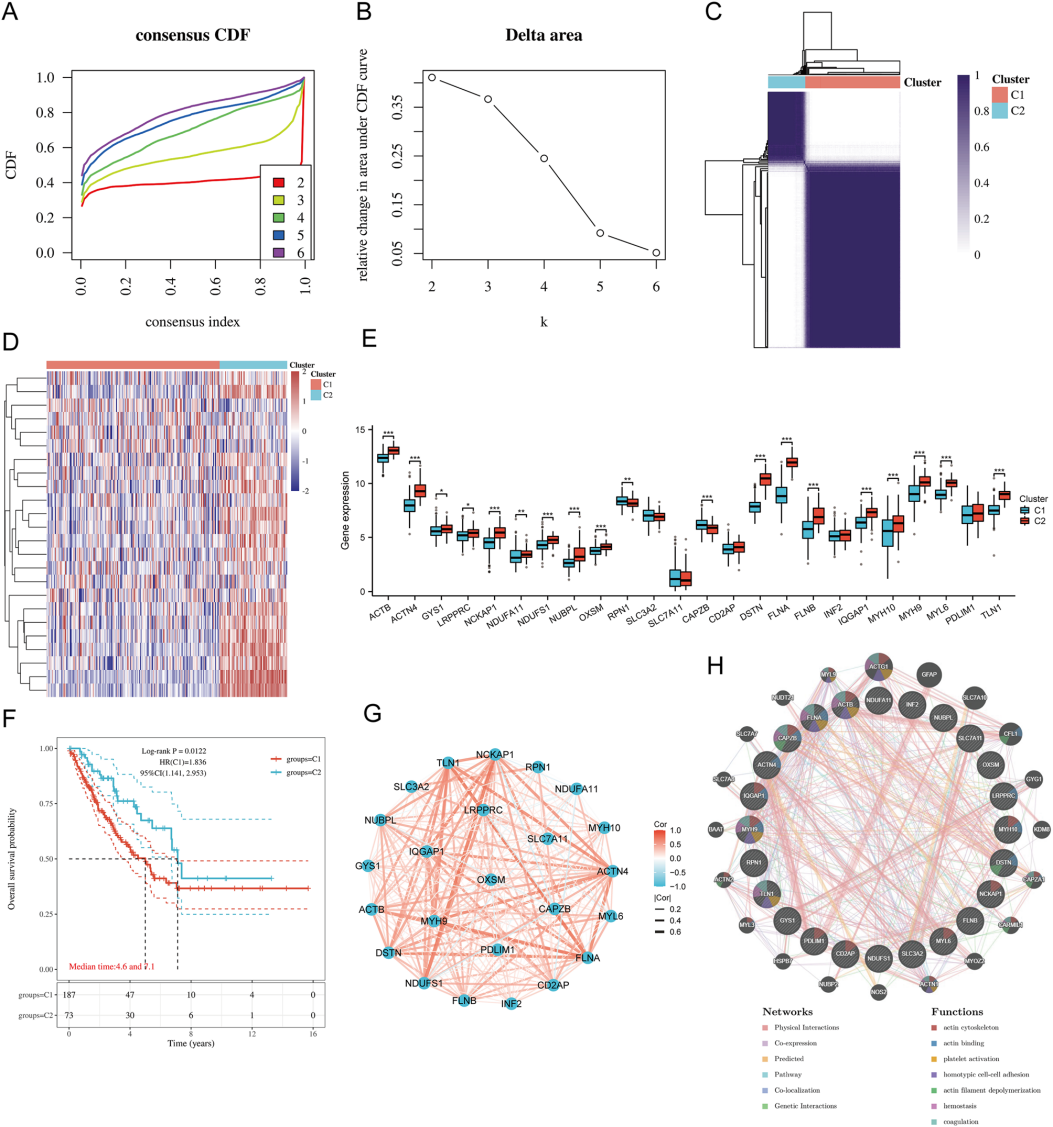
Common clusters were identified according to the expression of disulfidptosis-related genes (DRGs). (**A**) Cumulative distribution function (CDF) (k = 2–6); (**B**) The relative change of area under CDF curve (CDF Delta area) (k = 2–6); (**C**) Consensus clustering matrix (k = 2); (**D**) The heat map of DRG expression across different subtypes, where red color represents high expression and blue represents low expression. (**E**) Differential expression of DRGs between C1 and C2 subgroups in SARC samples. Moreover, the quartile ranges of the upper and lower representative values in the box, with the line representing the median value; (**F**) Kaplan–Meier survival analysis was conducted according to two clusters. (**G**) Pearson’s correlation analysis for the expression of 24 DRGs in SARC; (**H**) Gene Interaction Network.

The flowchart of the study has been illustrated in Supplementary Fig. S1. Consensus clustering helped categorize the 260 SARC samples in the TCGA database according to the expression levels of 24 DRGs in SARC. The tumor samples were classified into k (k = 2–6) distinct clusters. After the cluster analysis outcomes, the cluster count was two, signifying precise SARC patient segregation into two clusters (C1 and C2) (Fig. 1A–D). The TCGA-SARC dataset helped compare the expression levels of 24 DRGs between the C1 (n = 187) and C2 (n = 73) groups. The findings indicated notable variations within the expression levels of ACTB, ACTN4, GYS1, LRPPRC, NCKAP1, NDUFA11, NDUFS1, NUBPL, OXSM, RPN1, CAPZB, DSTN, FLNA, FLNB, IQGAP1, MYH10, MYH9, MYL6, and TNL1 genes between the two groups (*p* < 0.05) (Fig. 1E). Additionally, the Kaplan–Meier survival plot described that the overall survival (OS) of C1 patients was significantly inferior to C2 patients (Fig. 1F). Furthermore, most of the 24 DRGs in SARC samples showed a positive correlation (Fig. 1G). The findings from GeneMANIA indicated that DRG co-expressed in this network were linked with various processes, including actin cytoskeleton, actin binding, platelet activation, homotypic cell–cell adhesion, actin filament depolymerization, hemostasis, and coagulation (Fig. 1H).

### DEGs and functional enrichment analysis

**Figure 2:**
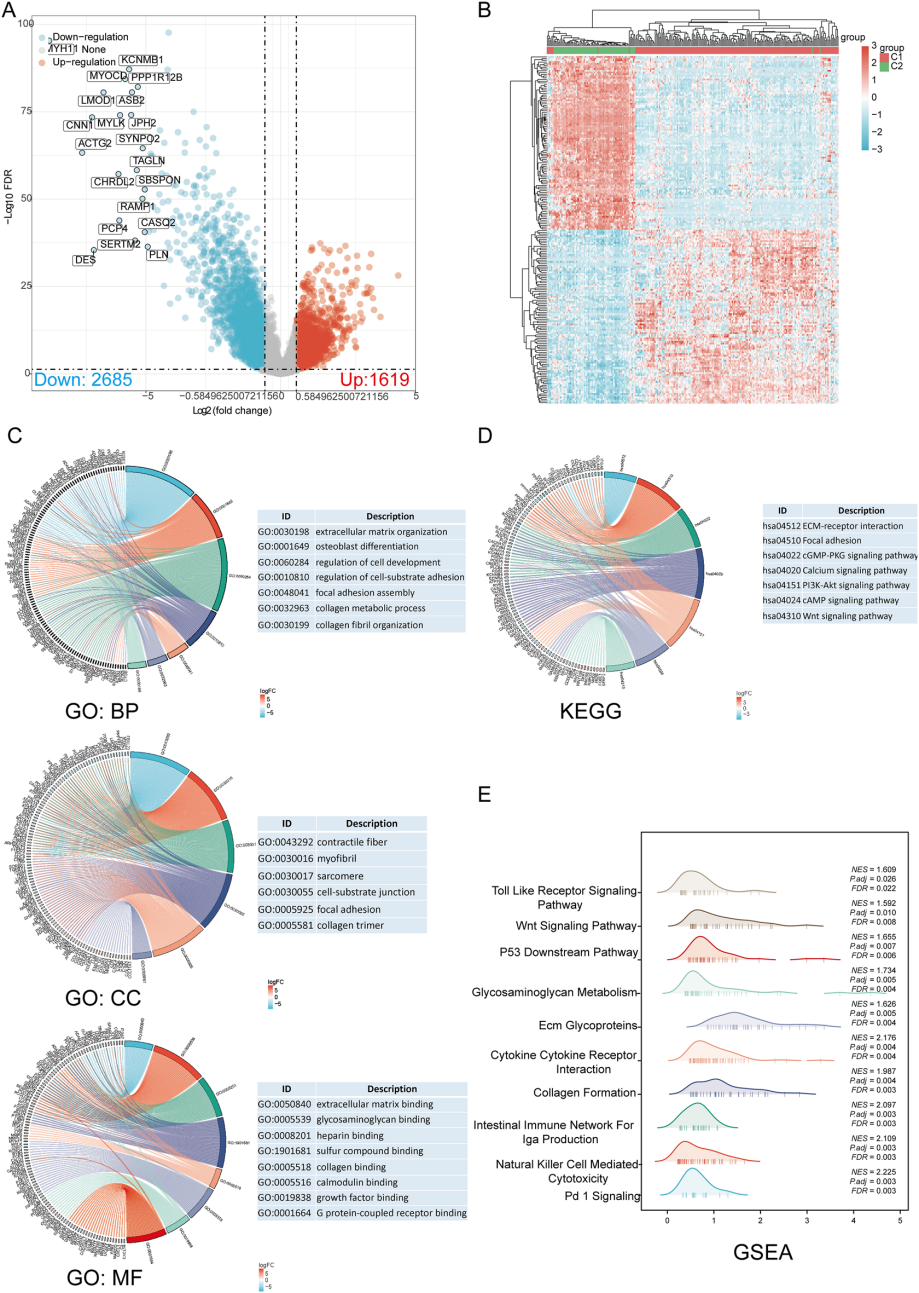
The screening of DEGs between DRG subtypes and functional enrichment analysis of DEGs. (**A**) The volcano plot of DEGs between C1 and C2 subtypes; (**B**) The DEG heatmap between C1 and C2 subtypes; (**C**, **D**) The enrichment analysis of GO and KEGG for DEGs; (**E**) The enrichment map from GSEA.

The DEGs identified between C1 and C2 subtypes involved 1,619 upregulated and 2685 downregulated genes. Then, a volcano map (Fig. 2A) and heat map (Fig. 2B) were constructed for the DEGs. The upregulated and downregulated DEGs were identified using GO and KEGG enrichment analysis. The GO analysis revealed that the DEGs were primarily enriched in extracellular matrix organization, osteoblast differentiation, cell-substrate adhesion regulation, collagen metabolic process, myofibril, cell-substrate junction, collagen trimer, extracellular matrix binding, glycosaminoglycan binding, sulfur compound binding, collagen binding, etc. (Fig. 2C). The enrichment analysis of DEGs in KEGG datasets revealed their enrichment in specific processes, such as ECM-receptor interaction, Focal adhesion, cGMP-PKG signaling pathway, Calcium signaling pathway, PI3K-Akt signaling pathway, cAMP signaling pathway, and Wnt signaling pathway, among others (Fig. 2D). GSEA pathway enrichment analyses revealed that DRG expression was closely linked with PD1 signaling, Natural killer cell mediated cytotoxicity, Intestinal immune network for IgA production, Collagen formation, Cytokine-cytokine receptor interaction, ECM glycoproteins, Glycosaminoglycan metabolism, P53 downstream pathway, Wnt signaling pathway, Toll-like receptor signaling pathway, B cell receptor signaling pathway, NOD-like receptor signaling pathway, etc. (Fig. 2E, Supplementary Table S2). These activated pathways increase tumor development and progression risk.

### Correlation analysis of genetic changes

**Figure 3:**
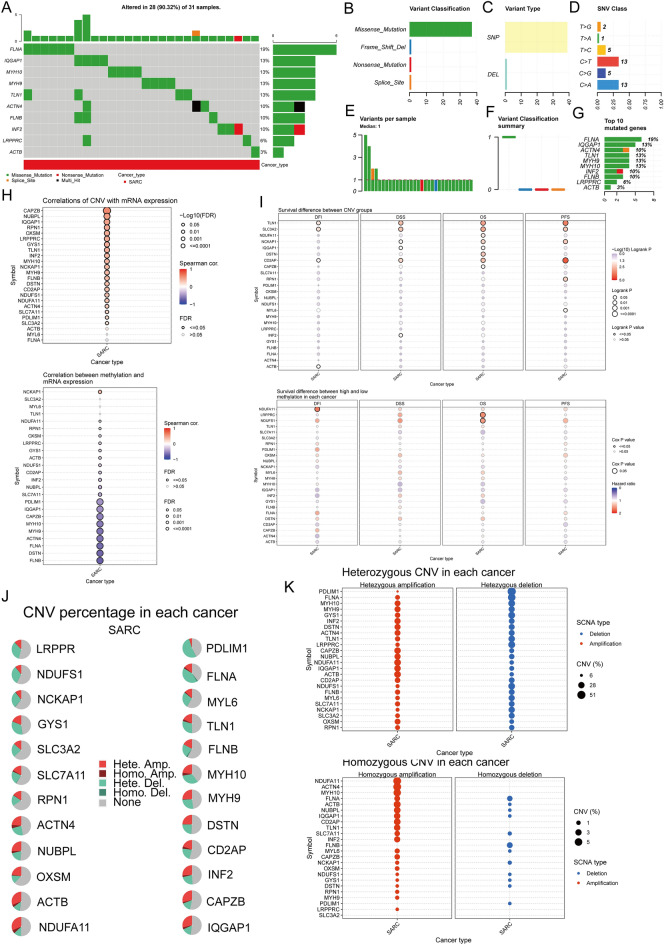
The correlation analysis of genetic alterations in DRGs. (**A**) The distribution of mutation types in the first 10 DRGs in SARC; (**B**–**D**) Variant classification, variant type, and SNV class. (**E**) The mutation burden per sample, (**F**) Variant classification summary. (**G**) The top 10 mutated genes within SARC. SNP, single nucleotide polymorphism. (**H**) The relationship between CNV, methylation, and DRG expression. Red depicts a positive correlation; blue indicates a negative correlation. The deeper the color, the higher the correlation index. Bubble size means the FDR (False Discovery Rate). (**I**) The correlation between DRGs CNV, methylation, and survival rates (DFI, DSS, OS, and PFS) in SARC patients. (**J**) CNV pie chart distribution. (**K**) The CNV map and homo CNV profile of 24 DRGs in the SARC cohort. SNP, single nucleotide polymorphism; SNV, single nucleotide variation; CNV, copy number changes.

We examined SNV distribution on the graph using the GSCA database. The frequency of FLNA mutations was elevated. In the DRGs, Oncoplot depicted the SNV for the initial 10 genes. FLNA (19%) and IQGAP1 (13%) possessed the highest mutation frequency. This was followed by *ACTN4 (10%), TLN1 (13%), MYH9 (13%), MYH10 (13%), INF2 (10%), FLNB (10%), LRPPRC (6%), and ACTB (3%)* (Fig. 3A). Different categories helped classify mutations with the most significant portion linked with missense mutations (Fig. 3B). Simultaneously, SNPs occurred more frequently than deletions (Fig. 3C), with C > T and C > A being prevalent (Fig. 3D). Furthermore, the computation of base alterations per individual indicated that the median and highest count of genetic variations were 1 and 5 (Fig. 3E). The number of occurrences for each variant classification is displayed inside the box plot (Fig. 3F). Furthermore, due to the overall count of mutations and conducting separate calculations for multiple impacts, the initial 10 genes were reevaluated with mutations (Fig. 3G). CNV and methylation levels affect gene expression levels and prognosis. We analyzed the correlation between DRG CNV, methylation status, and mRNA, indicating a significant positive correlation between DRG CNV and mRNA expression. In contrast, the gene methylation level was negatively correlated with mRNA expression (Fig. 3H). Figure 3I indicates that CNV and methylation levels for some DRGs are significantly associated with poor prognosis among SARC patients. Subsequently, we analyzed 24 DRG CNV landscapes in SARC (Fig. 3J), indicating elevated heterozygosity deletion/amplification rates (Fig. 3K). CNV analysis demonstrated that DRGs had heterozygous amplification and extensive heterozygosity loss. In contrast, *NDUFA11, ACTN4,* and *MYH10* indicate high-level homozygosity amplification, and *FLNA* and *FLNB* depict high-level homozygosity loss.

### Prognostic value and gene expression

**Figure 4:**
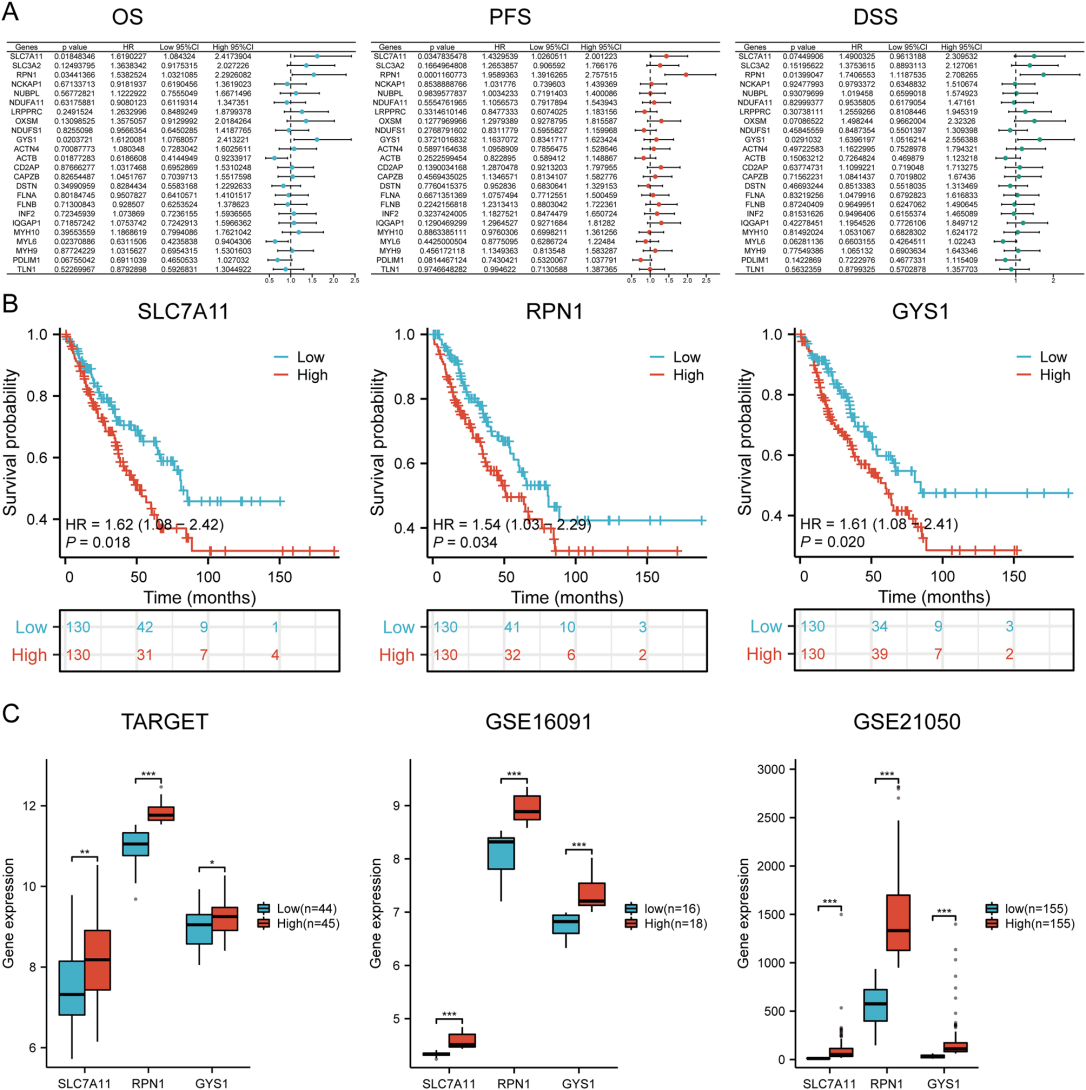
The prognostic value and gene expression. (**A**) The analysis of three prognostic DRGs from univariate Cox regression analysis plots; (**B**) The prognostic value of three DRGs (SLC7A11, RPN1, and GYS1) in high and low expression groups across SARC patients; (**C**) The mRNA expression of prognostic DRGs within TARGET and GEO dataset.

We identified three genes with prognostic value (*SLC7A11, RPN1,* and *GYS1*) using univariate Cox analysis and visualized using forest plot, including OS, PFS, and DSS (Fig. 4A). As shown in Fig. 4B, the OS rate of SARC patients with high expression levels of *SLC7A11* (log-rank *p* = 0.0185, HR = 1.619 (1.084–2.417)), *RPN1* (log-rank *p* = 0.0344, HR = 1.538 (1.032–2.293)), and *GYS1* (log-rank *p* = 0.0204, HR = 1.612 (1.077–2.413)) possessed a lower survival rate. Therefore, high *SLC7A11, RPN1,* and *GYS1* expressions are prognostic factors among SARC patients. Furthermore, we validated the expression levels of prognostic DRGs (*SLC7A11, RPN1,* and *GYS1*) through the Target and GEO databases. Compared to the low-expression group, the prognostic DRG expression levels in the high-expression group were significantly upregulated (*p* < 0.05) (Fig. 4C). Therefore, high expression of *SLC7A11, RPN1,* and *GYS1* could be a prognostic factor in SARC patients.

### Establishing a prognostic risk model

**Figure 5:**
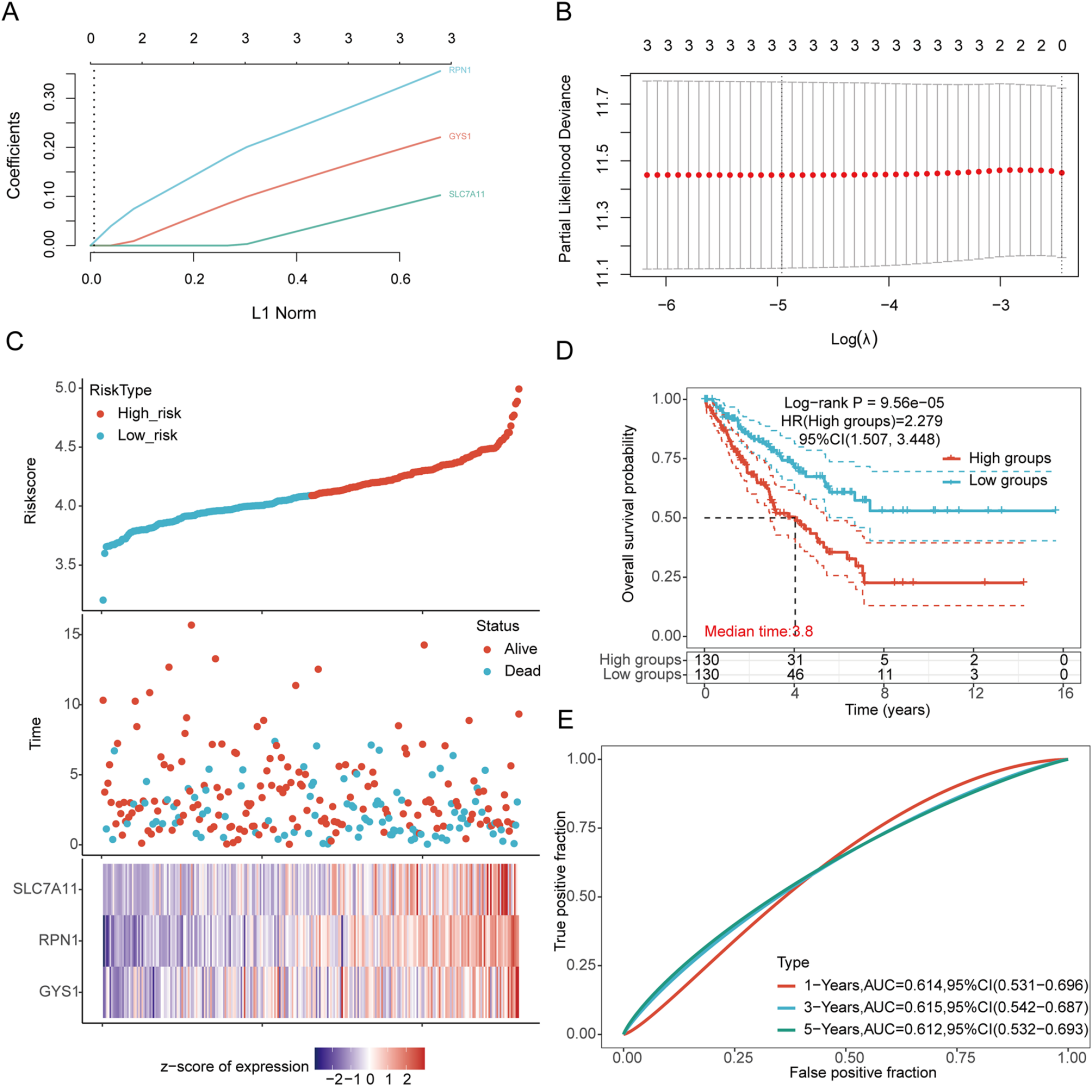
Construction of a prognostic model with the help of DRGs in SARC tissue. (**A**) LASSO coefficient profiles of three DRGs associated with the overall survival of SARC; (**B**) LASSO regression with tenfold cross-validation obtained three prognostic genes that error is within one standard error of the minimum (lambda.1se). A lambda value of 0.007 was chosen (lambda.min) according to tenfold cross-validation; (**C**) Distribution of risk score, survival status, and expression of prognostic DRGs in SARC patients; (**D**) Overall survival curve of SARC patients in high/low-risk groups; (**E**) Time-dependent ROC curve of sensitivity and specificity for the three DRGs in predicting the OS of patients for 1-, 3-, and 5-year.

We conducted LASSO Cox regression analysis to develop an OS prognosis model using the three prognostic DRGs and the expression profiles of possible prognostic biomarkers (Fig. 5A,B). To calculate the risk score for OS among SARC patients, the following formula was used: Riskscore = (0.0916) × SLC7A11 + (0.3376) × RPN1 + (0.2079) × GYS1. Based on the risk score, TCGA-SARC patients were categorized into two groups in the training cohort. The risk score distribution, survival status, and expression level of the three DRGs are represented in Fig. 5C,D. With the increase in risk score, the risk of death increased, and survival time decreased (Fig. 5C). The Kaplan–Meier curve indicated that SARC patients with high-risk scores had lower OS rates than those with low-risk scores [median time = 3.8 years, log-rank *p* = 9.56e−05, HR = 2.279 (1.507, 3.448)] (Fig. 5D). The AUCs for 1-, 3-, and 5-year ROC curves were 0.614 (95% CI 0.531–0.696), 0.615 (95% CI 0.542–0.687), and 0.612 (95% CI 0.532–0.693), respectively (Fig. 5E). The same analysis was conducted for DSS, which showed that Riskscore = (0.0307) × SLC7A11 + (0.3545) × RPN1 + (0.1558) × GYS1. The DSS of patients with high SARC expression was lower than that of low-expression patients [median time = 5.2 years, log-rank *p* = 0.000753, HR = 2.182 (1.386, 3.434)]. The AUCs for 1-, 3-, and 5-year ROC curves were 0.614 (95% CI 0.519–0.709), 0.617 (95% CI 0.541–0.693), and 0.6 (95% CI 0.515–0.685), respectively (Supplementary Supplementary Fig. S2). Therefore, the outcomes of the risk-scoring model associated with disulfidptosis depicted a notable correlation with the survival rate in SARC individuals.

### Internal and external validation of the expression patterns of the three prognostic genes

Risk scores were computed for every patient in the validation sets 1 and 2 of TCGA to ascertain the predictive significance of the three gene characteristics. Subsequently, the patients were categorized into high-risk and low-risk groups, with the median as the dividing point. The allocation of risk score, survival time, and expression of DRGs in every SARC patient was evaluated (Supplementary Fig. S3A,B). Patients in the high-risk group exhibited significantly poorer overall survival (OS) than those in the low-risk group, as demonstrated in Supplementary Fig. S3C,D of the validation set. In TCGA validation set 1, the AUC for 1-year, 3-year, and 5-year OS was 0.621 (95% CI 0.473–0.770), 0.612 (95% CI 0.481–0.743), and 0.623 (95% CI 0.486–0.760), respectively (Supplementary Fig. S3E). Similarly, the AUC for 1-year, 3-year, and 5-year OS in TCGA validation set 2 was0.611 (95% CI 0.479–0.744), 0.612 (95% CI 0.493–0.731), and 0.604 (95% CI 0.468–0.740), respectively (Supplementary Fig. S3F). DRG accuracy in predicting OS was high in TCGA validation sets 1 and 2. Subsequently, the ICGC (validation cohort) findings aligned with the outcomes observed in the TCGA cohort. Supplementary Fig. S3G depicts the distribution of risk scores, survival times, and DRG expression in every SARC patient. The overall survival OS of patients in the high-risk category was significantly lower than those in the low-risk category (*p* = 0.027, HR = 2.39 (1.11, 5.16)) as shown in Supplementary Fig. S3H. Supplementary Fig. S3I depicts that the AUC for 1-year, 3-year, and 5-year OS was (95% CI 0.363–0.958), 0.562 (95% CI 0.397–0.727), and 0.703 (95% CI 0.563–0.843), respectively. Furthermore, to validate the evaluation capacity of the model, we conducted survival analysis for 1-year, 3-year, and 5-year overall survival (OS) using the TCGA and ICGC cohorts combined as a whole. We plotted ROC curves to assess the predictive value of the combined prognostic features. The area under the curve (AUC) for 1-year, 3-year, and 5-year OS was 0.668 (95% CI 0.575–0.7606), 0.665 (95% CI 0.5833–0.7468), and 0.712 (95% CI 0.63–0.7936), respectively (Supplementary Fig. S4A). Kaplan–Meier survival curves for the high-risk and low-risk groups showed a significant association between high-risk group and poorer OS (*p* < 0.001, HR = 2.63 (1.68–4.13), Supplementary Fig. S4B). Comparing the AUC of the ROC curves for the three features, the combined cohort performed the best in terms of sensitivity and specificity, with an AUC of 0.712 (5-year) (Supplementary Fig. S4C). These findings support the previous results, indicating that the combined model provides more accurate prognostic assessment for SARC patients. In conclusion, these findings validate the efficiency of our risk assessment model. The three gene characteristics can predict the survival rate in SARC. The three gene characteristics can predict the survival rate in SARC. Taken together, these results confirm the validity of our risk score model and that DRG prognostic signature can predict OS in SARC.

### Building of a predictive nomogram

**Figure 6:**
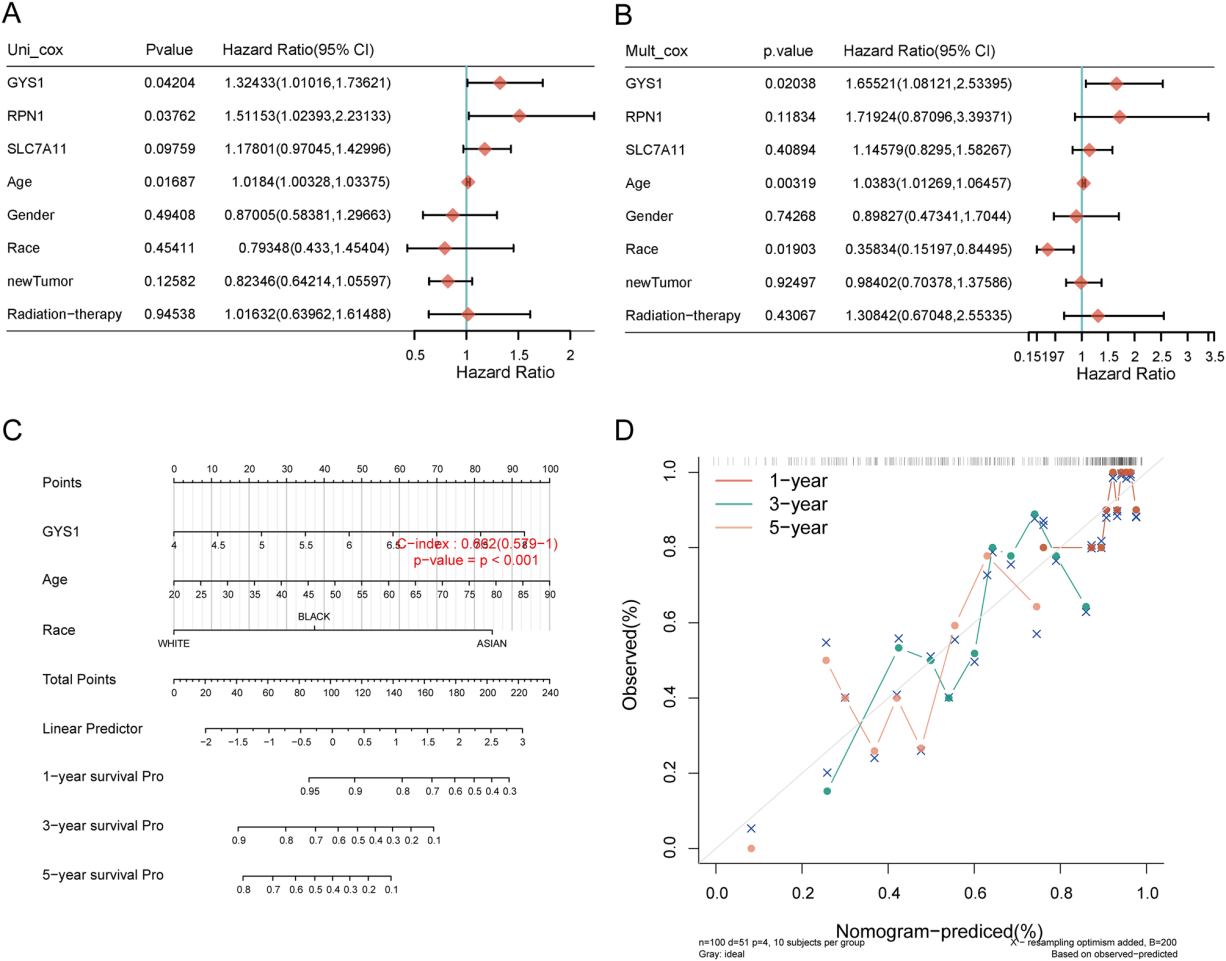
Predictive nomogram construction. (**A**, **B**) Hazard ratios and *p* values of the constituents associated with univariate and multivariate Cox regression analysis based on the clinical information and prognostic DRGs in SARC; (**C**) A nomogram to predict 1-, 3-, and 5-year OS of SARC patients; (**D**) The calibration curve of OS nomogram model within the discovery group. The diagonal dotted line characterizes the ideal nomogram.

A prognostic nomogram is developed for estimating the survival probability. According to univariate and multivariate regression analysis, the prognosis of SARC patients was affected by the expression of GYS1, age, and race (Fig. 6A,B). The results from the prognostic nomogram depict that the 1-, 3-, and 5-year overall survival (OS) [C-index 0.662 (0.579–1), *p* < 0.001] (Fig. 6C,D), disease-specific survival (DSS) [C-index 0.678 (0.594–1), *p* < 0.001] (Supplementary Fig. S5A–D) were precisely forecasted within the developed nomogram compared to the ideal model.

### Association between riskscore and clinical pathological features

**Figure 7:**
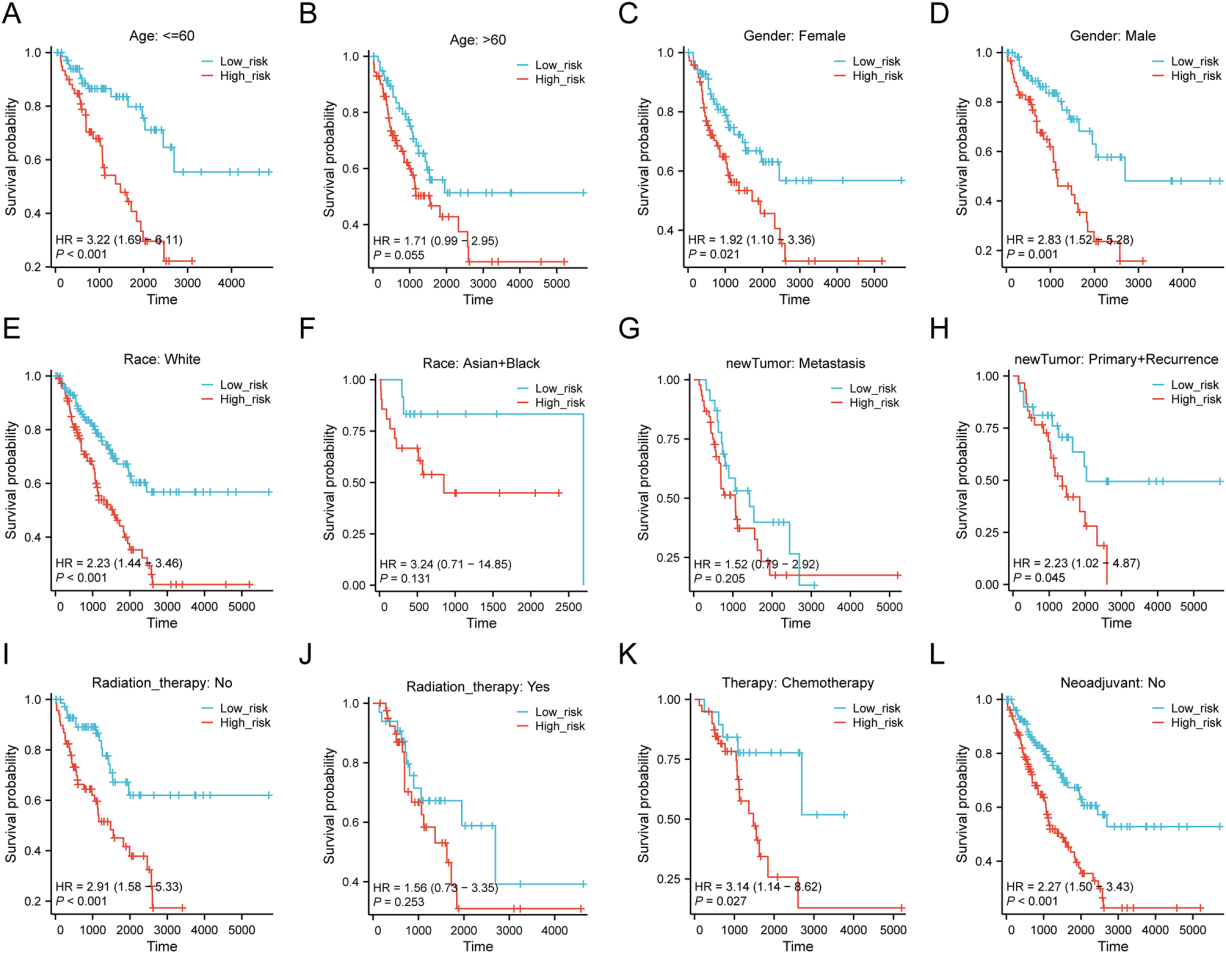
The survival curve of high-risk and low-risk patients across different subgroups of SARC, including age ≤ 60 (**A**), age > 60 (**B**), Female (**C**), male (**D**), White (**E**), Asian + Black (**F**), Metastasis (**G**), Primary + Recurrence (**H**), Radiation N0 (**I**), Radiation Yes (**J**), Chemotherapy Therapy (**K**), and Neoadjuvant N0 (**L**).

Based on the prognostic models, we explored survival analysis of clinical pathological features between high-risk and low-risk groups (Supplementary Table S3). The subgroup survival analysis revealed that the high-risk group significantly affected the overall survival time of age <  = 60 (*p* < 0.001, HR = 3.22 (1.69–6.11)), female (*p* = 0.021, HR = 1.92 (1.10–3.36)), male (*p* = 0.001, HR = 2.83 (1.52–5.28)), White (*p* < 0.001, HR = 2.23 (1.44–3.46)), Primary + Recurrence (*p* = 0.045, HR = 2.23 (1.02–4.87)), radiation N0 (*p* < 0.001, HR = 2.91 (1.58–5.33)), chemotherapy (*p* = 0.027, HR = 3.14 (1.14–8.62)), and neoadjuvant N0 (*p* < 0.001, HR = 2.27 (1.50–3.43)). However, age > 60 (*p* = 0.055, HR = 1.71 (0.99–2.95)), Asian + Black (*p* = 0.131, HR = 3.24 (0.71–14.85)), metastasis (*p* = 0.205, HR = 1.52 (0.79–2.92)), and radiation Yes (*p* = 0.253, HR = 1.56 (0.73–3.35)) were not significantly correlated with the overall survival time of SARC patients (Fig. 7A–L). These findings demonstrate that these variables significantly impact the prognosis of individuals with SARC and must be considered while formulating treatment approaches.

### Prognostic DRGs interfere with immune cell infiltration in SARC

**Figure 8:**
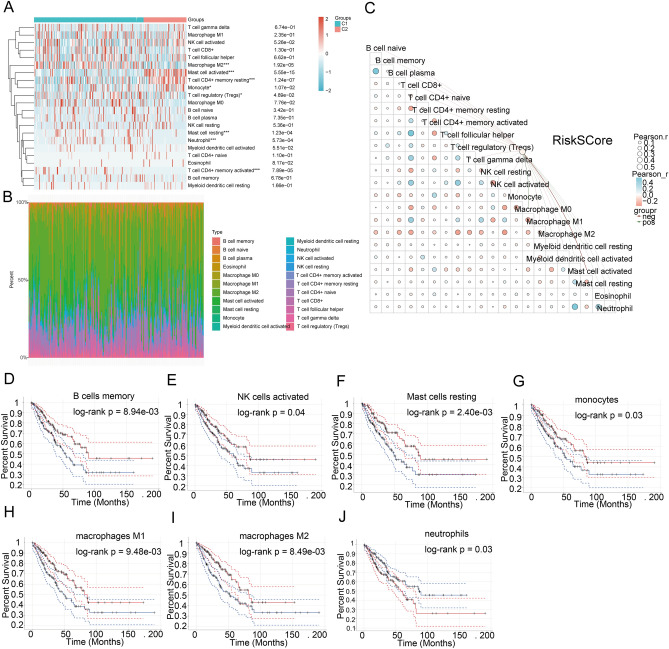
The relationship between the expression level of DRGs and immune infiltration within the tumor microenvironment. (**A**, **B**) Immune score comparison between C1 and C2 subtypes in TCGA (CIBERSORT); the abscissa indicates the type of immune cell infiltration, and the ordinate characterizes the distribution of the immune infiltration score across different groups; (**C**) The correlation analysis between Riskscore and Immunocore (CIBERSORT); (**D**–**I**) The relationship between the immune cell infiltration level and survival rate, such as B.cells.memory (**D**), NK.cells.activated (**E**), Mast.cells.resting (**F**), Monocytes (**G**), Macrophages.M1 (**H**), Macrophages.M2 (**I**), and Neutrophil (**J**).

We used six algorithms to observe the differences in immune cells between the C1 and C2 subtypes of SARC samples. Among them, the CIBERSORT algorithm revealed significant differences in T cell gamma delta, NK cell activated, T cell CD8 + , T cell follicular helper, Macrophage M2, T cell CD4 + memory resting, T cell regulatory (Tregs), Myeloid dendritic cell activated, and B cell memory between the two subtypes (Fig. 8A,B). Furthermore, the correlation analysis between Riskscore and immunological scores using the CIBERSORT algorithm indicated a significant association between Riskscore and various immune cell populations. Riskscore was negatively linked with B cell naive (*p* = 0.030, cor =  − 0.135), B cell memory (*p* = 0.022, cor =  − 0.142), T cell CD8 + (*p* = 0.034, cor =  − 0.132), NK cell activated (*p* = 0.041, cor =  − 0.127), Myeloid dendritic cell resting (*p* = 0.023, cor =  − 0.141), and Mast cell activated (*p* < 0.001, cor =  − 0.287). Moreover, Riskscore was positively correlated with Macrophage M0 (*p* < 0.001, cor = 0.234) and Macrophage M2 (*p* = 0.012, cor = 0.156) (Fig. 8C). Similarly, in the TIMER, xCell, MCP-counter, quantTIseq, and EPIC algorithms, significant differences were also observed in immunologic infiltration score distribution between C1 and C2 subtypes (Supplementary Fig. S6A–E). Furthermore, there was a correlation between Riskscore and various immune cell populations (Supplementary Fig. S7A–E). Thus, immune infiltration may affect patient prognosis. Therefore, we conducted a survival analysis of different immune cell types indicating that high levels of B cell memory, NK cells activated, Mast cells resting, monocytes, macrophages M1, macrophages M2, and neutrophils were connected with good prognosis (Fig. 8D–J).

According to the ssGSEA method, a notable variation in immune cell infiltration was observed when comparing the high and low-expression groups of *SLC7A11, RPN1,* and *GYS1* (Supplementary Fig. S8A). Correlation analysis demonstrated that SLC7A11 expression positively correlated with Th2 cells, Macrophages, and T helper cells while negatively correlated with pDC, Eosinophils, NK cells, and CD8 T cells. On the other hand, RPN1 expression depicted a positive correlation with Th2 cells, Macrophages, and Neutrophils but a negative correlation with NK cells, pDC, Tem, and Mast cells. Additionally, GYS1 expression positively correlated with Tcm, NK CD56bright cells, and NK cells while negatively associated with Th1 cells, T cells, DC, and iDC (Supplementary Fig. S8B). These findings indicated a specific correlation between DRGs and tumor immune infiltration.

### Potential value of DRGs in the immunotherapy of SARC patients

**Figure 9:**
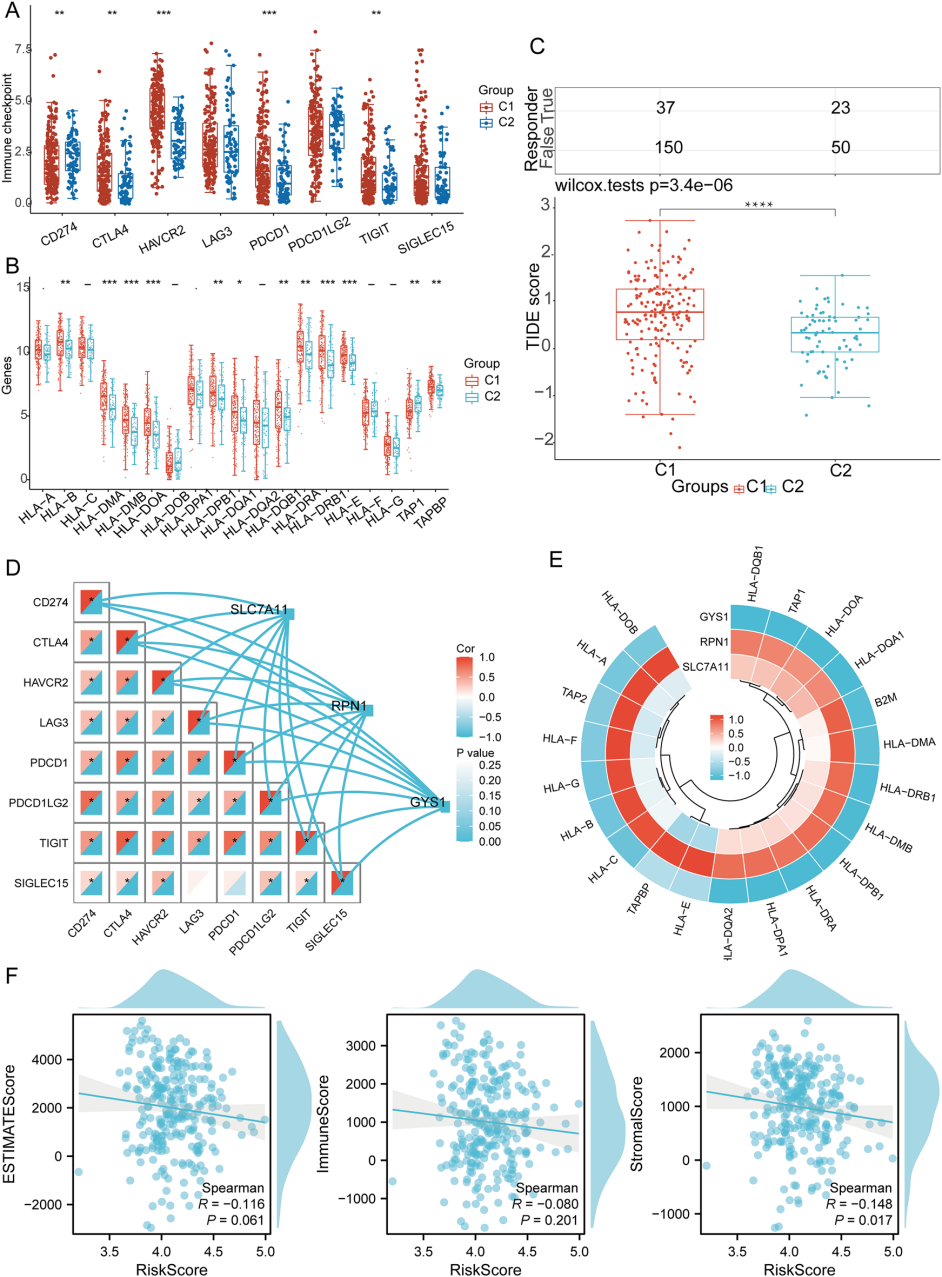
The correlation between prognostic DRG expression and immunogenicity. (**A**) The expression distributions of eight immune checkpoint-associated genes in SARC subtypes; (**B**) The differences in HLA members between C1 and C2 subtypes; (**C**) Different reactions of C1 and C2 subtypes to immune checkpoint blocking; (**D**) The correlation between the prognostic DRGs in SARC and immune checkpoint-related genes; (**E**) The correlation between the prognostic DRGs across SARC and HLA members; (**F**) Correlation between the risk scores and ESTIMATE in SARC.

We discussed the differences in expression between the two subtypes according to the eight immune checkpoint-related genes. The results indicated significant differences in expression levels of *CD274* (*p* = 0.006), *CTLA4* (*p* = 0.002), *HAVCR2* (*p* < 0.001), *TIGIT* (*p* = 0.002), and *PDCD1* (*p* < 0.001) between the two subtypes in group C1. Moreover, the expression levels of *CTLA4, HAVCR2, TIGIT,* and *PDCD1* were all higher than those in group C2 with a statistical difference (Fig. 9A). Then, we explored the expression distribution of HLA members in groups C1 and C2. The results depicted that HLA members (*HLA-B, HLA-DMA, HLA-DMB, HLA-DOA, HLA-DPB1, HLA-DQA1, HLA-DQB1, HLA-DRA, HLA-DRB1, HLA-E, TAP1, TAPBP*) were highly expressed in group C1 compared to C2 (Fig. 9B). In addition, the C2 subtype responded better to immune checkpoint blocking than C1 (Fig. 9C). Furthermore, an additional study was conducted on the relationship between prognostic DRG expression and immune checkpoints and HLA members in the TCGA database. The results showed that *SLC7A11* was positively associated with *SIGLEC15* (*p* < 0.001, cor = 0.232), while *LAG3* was negatively correlated (*p* = 0.020, cor =  − 0.143). *GYS1* was negatively correlated with *PDCD1LG2* (*p* = 0.020, cor =  − 0.144), *PDCD1* (*p* = 0.035, cor =  − 0.130), *CTLA4* (*p* = 0.006, cor =  − 0.168), and *TIGIT* (*p* < 0.001, cor =  − 0.205). DRG expression is significantly associated with most HLA members in SARC (Fig. 9D,E). Considering the above analysis of differences in immune cell infiltration, we further analyzed the correlation between Riskscore and three ESTIMATE. The analysis showed that there was a significant negative correlation between riskscore and StromalScore (*p* = 0.017, cor =  − 0.148), but no significant correlation with ESTIMATEScores (*p* = 0.061, cor =  − 0.116) and ImmuneScores (*p* = 0.201, cor =  − 0.080) (Fig. 9F). The results depicted a significant correlation between DRGs and tumor immune infiltration, becoming a potential immunotherapy target.

### TMB, MSI, and mRNAsi analysis

**Figure 10:**
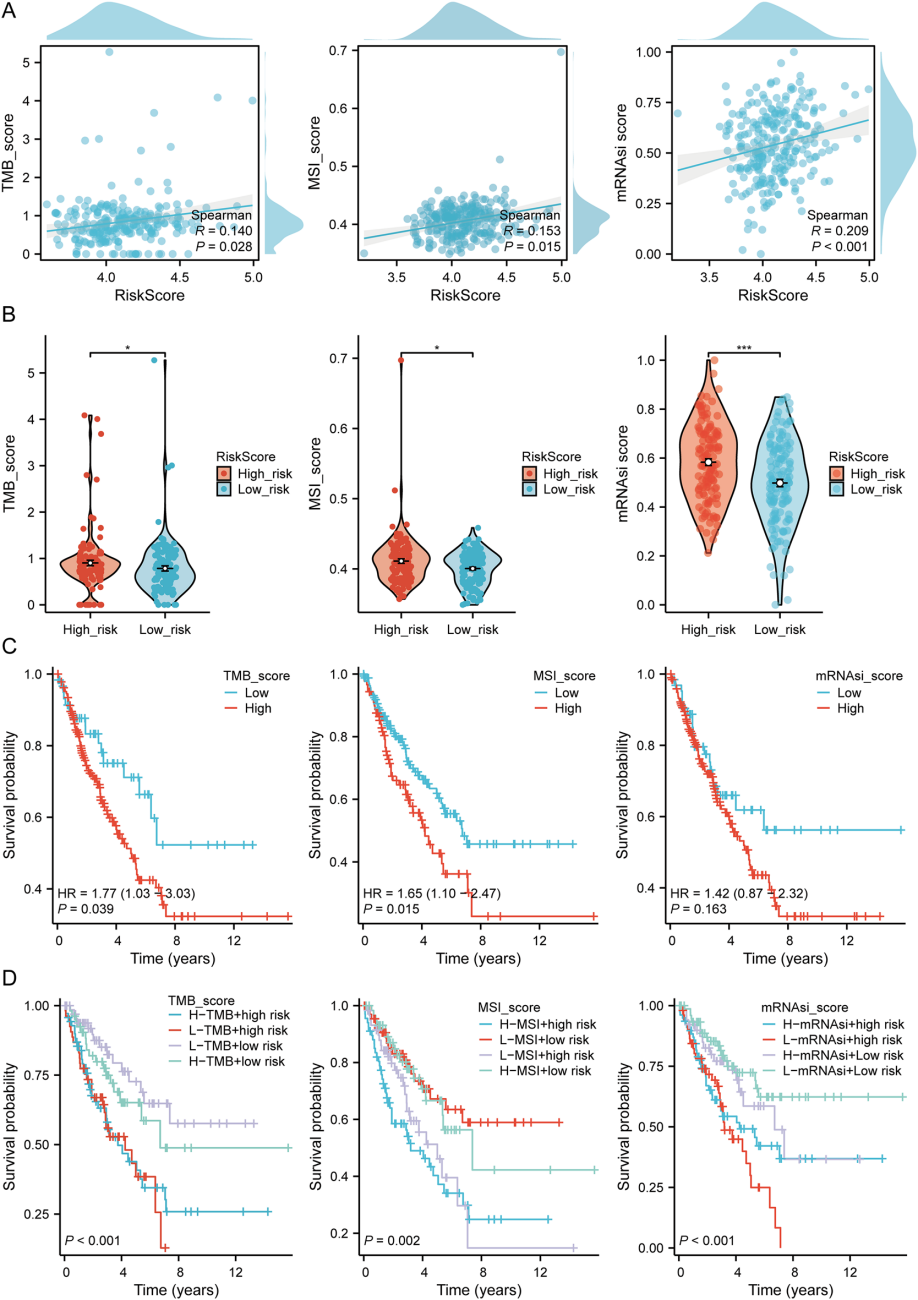
TMB, MSI, and mRNAsi analysis. (**A**) TMB, MSI, and mRNAsi score differences between the high and low riskscore within SARC; (**B**) Riskscore correlation according to the prognostic model with TMB, MSI, mRNAsi score in SARC patients; (**C**) The Kaplan–Meier curves of high and low TMB, MSI, mRNAsi groups in SARC; (**D**) The Kaplan–Meier curves of four groups classified by risk score and TMB, MSI, mRNAsi in SARC.

We evaluated the correlation between riskscore and TMB, MSI, ESTIMATE, and mRNAsi to determine the role of DRGs in TME during immune mechanisms and responses. The results indicated that the TMB, MSI, and mRNAsi scores of high-risk groups were significantly higher than those of low-risk groups (Fig. 10A). Moreover, riskscore were positively correlated with TMB, MSI, mRNAsi (Fig. 10B). We further performed survival analysis combining riskscores with TMB, MSI, and mRNAsi. The results revealed that patients with high TMB (*p* = 0.039, HR = 1.77 (1.03–3.03)), MSI (*p* = 0.015, HR = 1.65 (1.10–2.47)), and mRNAsi (*p* = 0.163, HR = 1.42 (0.87–2.32)) scores had a shorter OS (Fig. 10C). We divided patients into four subgroups and performed survival assessment to investigate the combined impact of riskscores and TMB on SARC patients survival. The OS was better in the low TMB + low riskscore than the high TMB + high riskscore (*p* < 0.001). Similarly, patients in the high MSI + high-risk group had a worse prognosis than those in the low MSI + low-risk group (*p* = 0.002). The OS of patients in the low mRNAsi + low-risk group was better than those in the high mRNAsi + high-risk group (*p* < 0.001) (Fig. 10D). Therefore, high-risk groups could have an immune response and respond to immunotherapy.

### Drug sensitivity analysis

Finally, we explored the potential value of new therapeutic targets of *SLC7A11, RPN1,* and *GYS1*. Some drugs were selected from GDSC and CTRP databases, indicating a significant correlation between risk scores and drug sensitivity (Supplementary Fig. S9A). Among high-risk SARC, the sensitivity of BMS-754807, Lapatinib, and Nutlin.3a was significantly higher than that in the low-risk group. In contrast, Bortezomib, foretinib, and MG-132 possessed higher sensitivity in low-risk SARC (Supplementary Fig. S9B). The results of Pearson correlation analysis depicted that the expression levels of riskscore were positively associated with Lapatinib, Nutlin.3a, but negatively correlated with Bortezomib, foretinib, and MG-132 (Supplementary Fig. S9C). These drugs may become potential therapeutic options for SARC.

### Immunotherapy response analysis

Based on previous relevant literature and the analysis results mentioned above, we found that the expression of DRGs in SARC patients is closely associated with immunity. To further predict the role of DRGs in immune therapy response, we first utilized two external independent immune therapy cohorts, GSE111636 dataset and IMvigor210, to evaluate the performance of DRGs in predicting immune therapy response (Anti-PD-1/PD-L1/CTLA-4). Additionally, the AUC results further confirmed the accuracy of risk scoring in predicting immune response, with AUC values of 0.964 (95% CI 0.865–1.000) and 0.620 (95% CI 0.547–0.693), respectively (Supplementary Fig. S10A,B). The results showed that patients who responded to immune therapy in both cohorts exhibited higher-risk DRGs, indicating that patients in the higher-risk DRGs group are more likely to benefit from immune therapy. Furthermore, to validate the predictive role of DRGs risk scoring in immune therapy response in clinical tissue samples of SARC, we examined 24 advanced sarcoma patients receiving anti-PD-1/PD-L1 treatment. The results indicated that patients achieving complete or partial response (CR/PR) were more prevalent in the high-expression groups of prognostic DRGs and high-risk score groups of the prognostic model. ROC analysis also confirmed the efficacy of risk scoring in predicting responders to ICI therapy (Supplementary Fig. S10C,D). Finally, in the GSE91061 immune therapy cohort, the overall survival of low-risk patients was superior to that of high-risk patients (*p* = 0.012, HR = 1.90 (1.15–3.12) (Supplementary Fig. S10E). Therefore, DRGs risk scoring holds great potential in predicting immune therapy, and patients with high DRGs risk scores may be more sensitive to ICI therapy.

### Single-cell RNA data analysis

**Figure 11:**
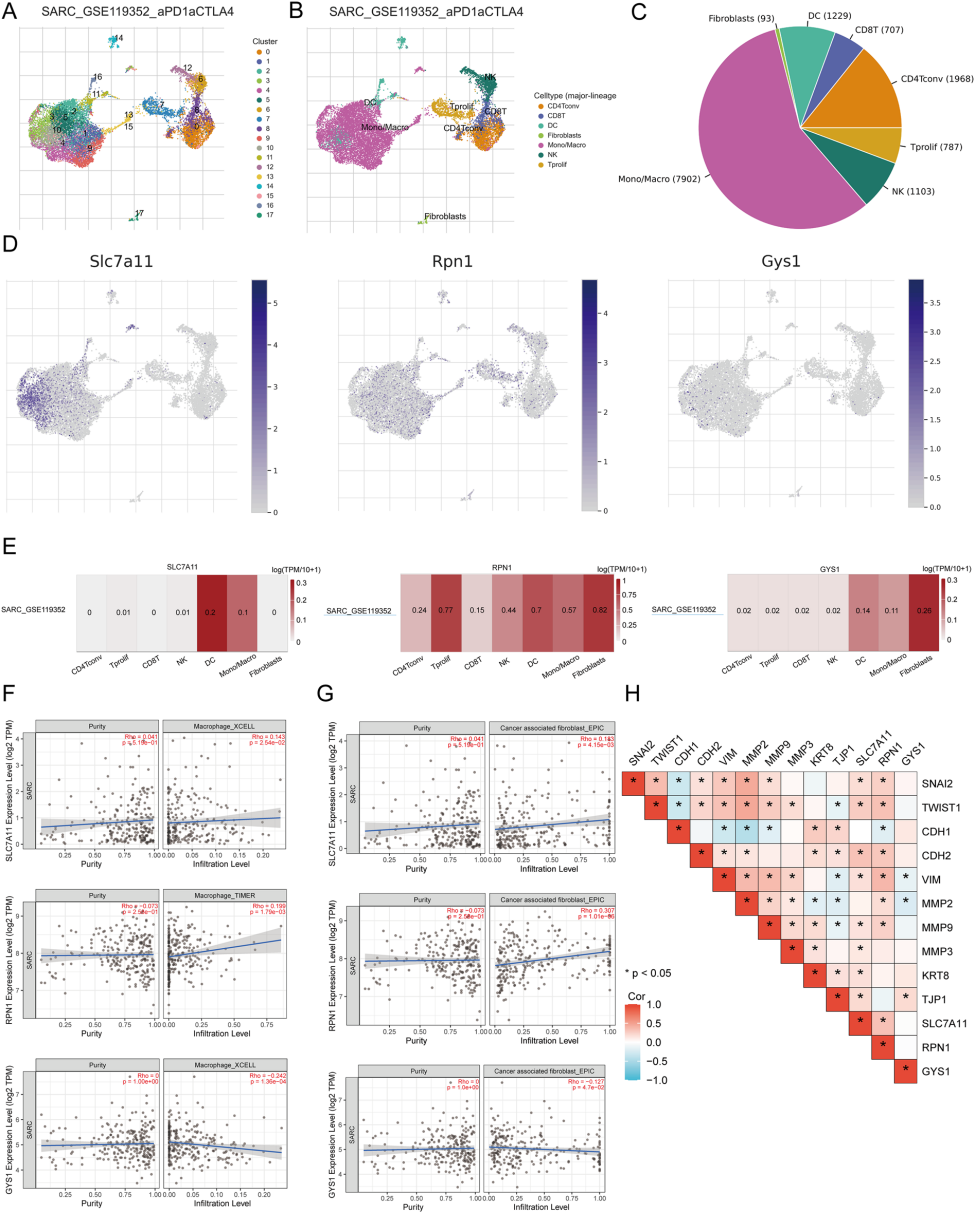
The expression of three prognostic DRGs across different immune cell types in SARC. (**A**) The cluster diagram of cell types in scRNA seq data. (**B**) The annotation of different immune cell lineages (SARC_GSE119352_aPD1aCTLA4) in SARC tissues; (**C**) The pie chart indicates the percentage of each cell. (**D**) The characteristic maps of three prognostic DRGs retrieved from scRNA-seq data; (**E**) The heat maps of three prognostic DRGs procured from scRNA-seq data; (**F**) The correlation between the expression of three prognostic DRG macrophages infiltration analyzed using TIMER2.0; (**G**) The correlation between the expression of three prognostic DRGs and CAF infiltration analyzed using TIMER2.0; (**H**) The correlation between the expression of three prognostic DRGs and EMT-associated markers.

Within the TISCH database, SARC_GSE119352_aPD1aCTLA4 was divided into 17 cell clusters and seven cell types, enabling the visualization of the distribution and number of different TME-related cells (Fig. 11A,B). The pie chart reveals that macrophages (Mono/Macro) are the most abundant in SARC_GSE119352_aPD1aCTLA4 (Fig. 11C). We utilized the SARC single cell GSE dataset (SARC_GSE119352_aPD1aCTLA4) to assess the expression levels of *SLC7A11, RPN1,* and *GYS1* on a single cell level (Fig. 11D). Single-cell RNA sequencing and fractionation involved Conventional CD4 T cells (CD4Tconv), proliferating T cells (Tprolif), CD8 T cells (CD8T), Natural killer cells (NK), Dendritic cells (DC), Monocytes or macrophages (Mono/Macro), and Fibroblasts (Fig. 11E). *SLC7A11, RPN1,* and *GYS1* were strongly expressed in fibroblasts, Mono/Macro, DC, and Tprolif. Simultaneously, immune infiltration analysis indicated a correlation between *SLC7A11, RPN1, GYS1* expression, and CAF, macrophages infiltration (Fig. 11F,G). Then, we analyzed the impact of prognosis DRG expression on EMT and observed the correlation between prognosis DRGs and EMT-related markers (SNAI2, TWIST1, CDH1, CDH2, VIM, MMP2, MMP9, MMP3, KRT8, and TJP1). The results indicated that prognosis DRGs were significantly linked with EMT marker genes (Fig. 11H), suggesting that prognosis DRGs-mediated EMT may promote fibroblast activation.

### Pan-RNA epigenetic modification-related gene expression

**Figure 12:**
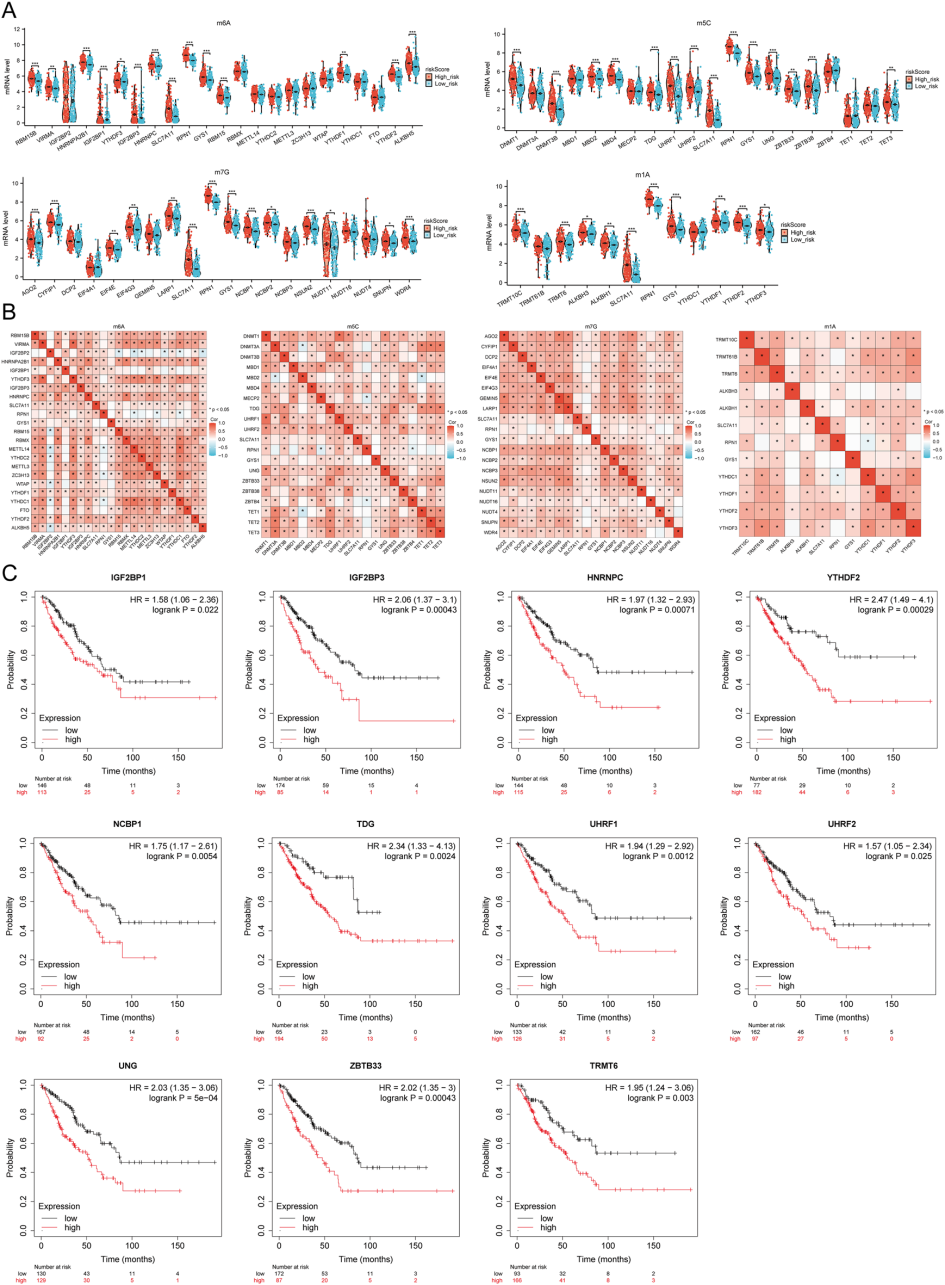
The correlation between the prognostic DRGs and pan-RNA epigenetic modification-related genes. (**A**) Differences in m 6 A, m 5 C, m 1A, and m7G among the high and low-risk groups in SARC; (**B**) The correlation between three prognostic DRGs and m6A, m5C, m1A, m7G-related genes within the TCGA-SARC cohort; (**C**) IGF2BP1, IGF2BP3, HNRNPC, YTHDF2, NCBP1, TDG, URF1, UHRF2, UNG, ZBTB33, and TRMT6 survival curves.

This study investigated whether DRG expression is linked with pan-RNA epigenetic modification by analyzing the differential expression of pan-RNA epigenetic modification-related genes among high and low-risk groups. The modification genes m6A, m5C, m1A, and m7G had significant differences between the two groups (*p* < 0.01). Among them, m6A, m5C, m1A, and m7G modification genes indicated high expression in the high-risk group (Fig. 12A). Moreover, the correlation between these three prognostic DRG expression and pan-RNA epigenetic modification-related gene expression was analyzed with the TCGA dataset. The results indicated that these three prognostic DRG expressions were significantly associated with m6A, m5C, m1A, and m7G modification genes (Fig. 12B). All three prognostic DRG expressions were positively linked with highly expressed *IGF2BP1, IGF2BP3, HNRNPC, YTHDF2, NCBP1, TDG, URF1, UHRF2, UNG, ZBTB33,* and *TRMT6*. Additionally, they were significantly associated with SARC prognosis (Fig. 12C), suggesting a close relation of DRG expression with DNA methylation modification in SARC.

### Prediction and verification of upstream key miRNA

Initially, the intersection of RNA22, TargetMiner, and miRWalk databases produced 665 sets of SLC7A11-miRNA pairs and 1215 sets of RPN1-miRNA pairs (Supplementary Fig. S11A). Thus, we theorized a negative association between the anticipated interactions of mRNA and miRNA depending on the traditional function of miRNA while inhibiting gene expression. To identify these potential miRNA expression correlations in SARC, we used Pan-cancer, a subproject in the ENCORI database. The findings depicted a notable inverse relationship between SLC7A11-hsa-miR-29c-3p and RPN1-hsa-miR-143-3p, as demonstrated in Supplementary Fig. S11B. In theory, miRNAs with a strong binding affinity to SLC7A11 and RPN1 are expected to be downregulated in SARC, leading to a negative prognosis. Hence, the predictive impact of these possible miRNAs in SARC was additionally confirmed by utilizing the Kaplan–Meier plotter database. The findings indicated that decreased levels of hsa-miR-29c-3p and hsa-miR-143-3p were associated with a notably unfavorable prognosis (Supplementary Fig. S11C). hsa-miR-29c-3p and hsa-miR-143-3p were validated as promising miRNAs with prognostic potential in SARC by merging the outcomes of inverse correlation and survival rate. The findings indicate that SLC7A11-hsa-miR-29c-3p and RPN1-hsa-miR-143-3p could potentially serve as the crucial pathway for the onset and progression of SARC while associating with patient prognosis.

### Prediction and validation of key miRNAs and potential LncRNAs

**Figure 13:**
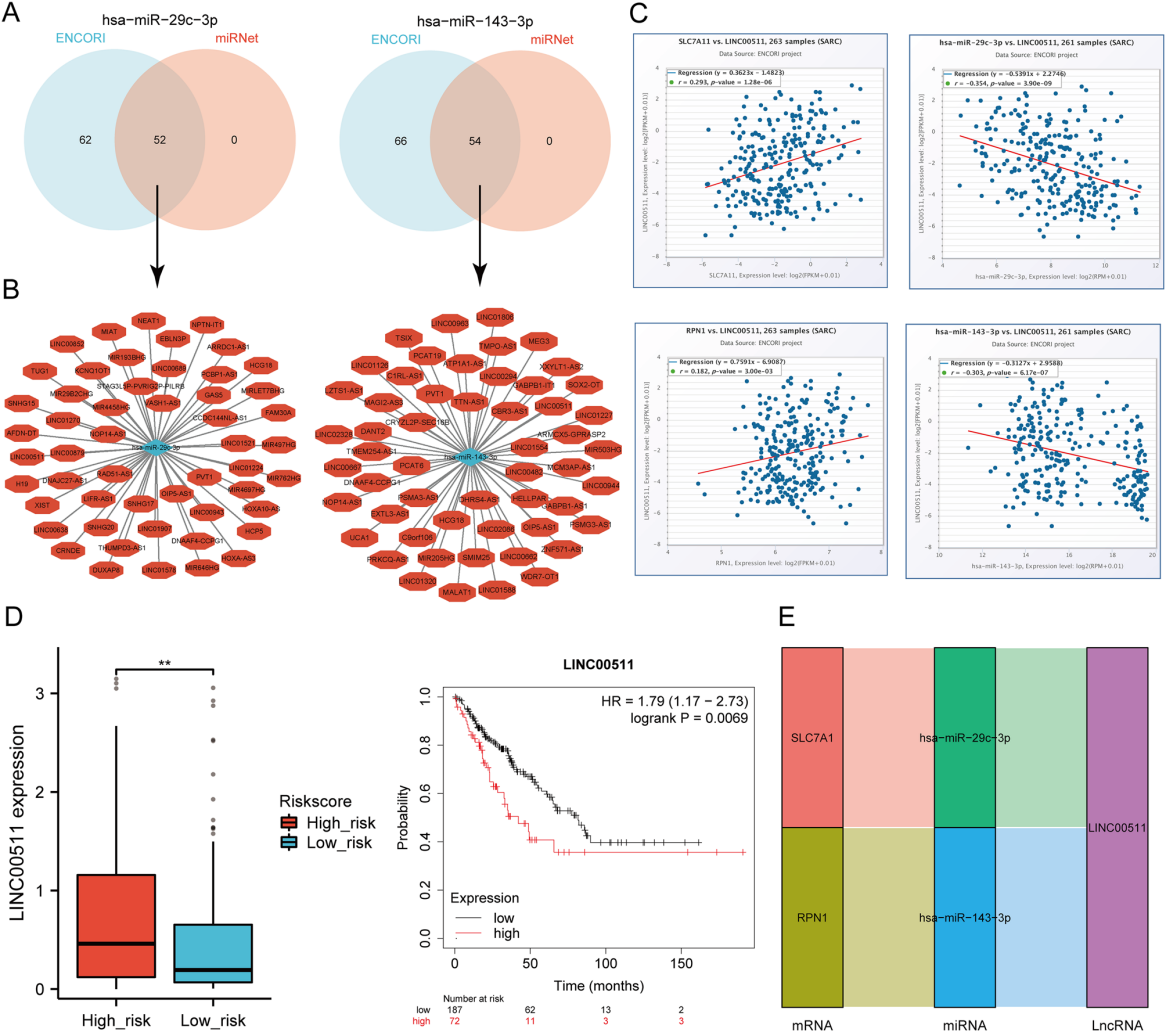
Screening of the LncRNA-miRNA-DRGs regulating axis in SARC. (**A**) The prediction of potential lncRNAs of hsa-miR-29c-3p and hsa-miR-143-3p using miRNet and ENCORI database; (**B**) The Cytoscape software helped build potential miRNA-lncRNA network; (**C**) The correlation of the one potential LncRNA(LINC00511) using hsa-miR-29c-3p, hsa-miR-143-3p, and SLC7A11 RPN1 in SARC; (**D**) Expression levels and prognostic values of the potential LncRNA(LINC00511) in SARC; (**E**) Triple regulatory networks of mRNA-miRNA-lncRNA impacting SARC prognosis.

We made a forecast of the miRNA's target upstream lncRNA to establish the miRNA-lncRNA axis. To anticipate lncRNAs that could be paired with hsa-miR-29c-3p and hsa-miR-143-3p, the ENCORI and miRNet databases were used. This analysis revealed a total of 52 lncRNAs targeting hsa-miR-29c-3p and 54 lncRNAs targeting hsa-miR-143-3p (Fig. 13A). To improve visualization, the Cytoscape software (Fig. 13B) was used to construct the network of miRNA-lncRNA regulation. The ceRNA hypothesis indicates that lncRNA can enhance mRNA expression via competitive binding to miRNA. Hence, lncRNA exhibited an inverse correlation with miRNA or a direct mRNA correlation. ENCORI database detected a correlation between lncRNAs and hsa-miR-29c-3p and hsa-miR-143-3p expressions. The analysis revealed that EBLN3P, LINC00943, and LINC00511 correlated significantly with hsa-miR-29c-3p and SLC7A11. In contrast, LINC01806, LINC01554, LINC00944, LINC00511, SMIM25, and MIR503HG showed significant association with hsa-miR-143-3p and RPN1 (Supplementary Table S4, Fig. 13C, and Supplementary Figs. S12, S13). Following this, the predictive significance of LncRNAs in SARC was evaluated using the Kaplan–Meier plotter, and the TCGA-SARC database helped measure the expression levels of the LncRNAs in SARC. The findings indicated a significant increase in the expression of LINC00511 in the high-risk group than in the low-risk group. Moreover, the elevated levels of LINC00511 expression were connected with an unfavorable prognosis for SARC patients (Fig. 13D). Thus, a significant mRNA-miRNA-lncRNA triple regulatory network linked with SARC prognosis was successfully identified. This network comprised two mRNAs (SLC7A11, RPN1), two miRNAs (hsa-miR-29c-3p, hsa-miR-143-3p), and one lncRNA (LINC00511) (Fig. 13E).

### Cellular experiments and clinical sample validation

**Figure 14:**
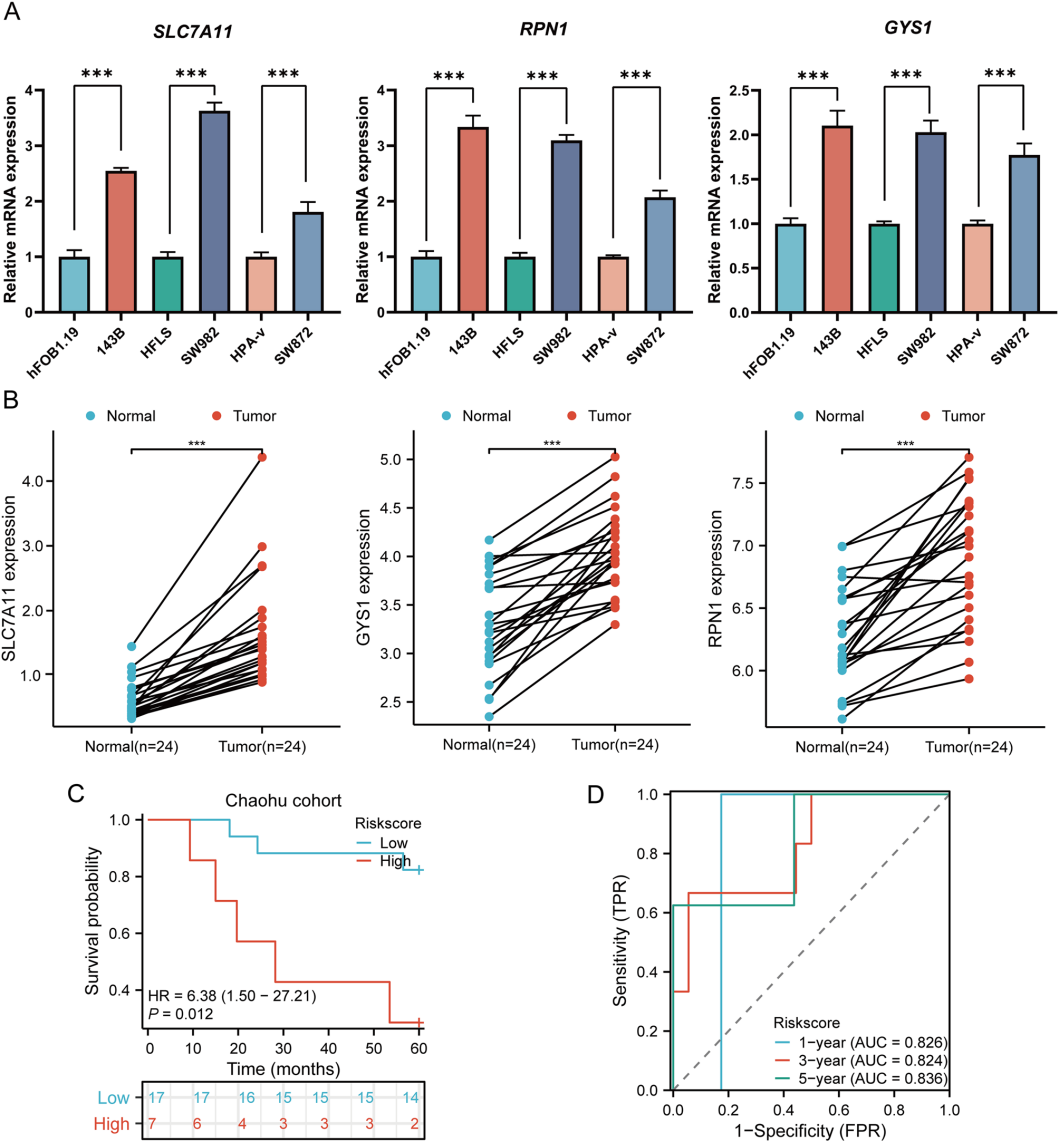
Cellular experiments and clinical sample validation. (**A**) The relative mRNA expressions of SLC7A11, RPN1, GYS1 in SARC cell lines and the corresponding normal cell lines. (**B**) The figures of qRT-PCR showed the relative expression of the three prognostic DRGs in normal tissues and SARC tissues. (**C**) Overall survival curve of HNSCC patients in high/low-risk groups; (**D**) Time-dependent ROC curve for 1-, 3-, and 5-year OS for DRGs.

In order to validate the analysis results from TCGA at the mRNA level, we conducted RT-qPCR validation to explore the mRNA expression of prognostic DRGs in SARC cell lines. Compared to their corresponding normal cell lines, SLC7A11, RPN1, and GYS1 exhibited significantly upregulated mRNA expression in sarcoma cell lines (143B, SW982, and SW872) (Fig. 14A). Furthermore, we employed RT-qPCR to detect the expression of the three prognostic DRGs in 24 SARC tissues and adjacent non-tumor tissues. Similar to the cellular expression levels, we found that the expression of SLC7A11, RPN1, and GYS1 was significantly upregulated in SARC tissues compared to normal tissues (Fig. 14B). Lastly, based on the prognostic model constructed from the TCGA-SARC dataset, we validated the predictive performance of this model using clinical tissue samples from our institution. Utilizing the aforementioned formula to calculate the risk score, patients were stratified into high-risk and low-risk groups. Survival analysis revealed that patients with higher risk scores had shorter overall survival compared to those with lower risk scores (*p* = 0.012, HR = 6.38 (1.50–27.21), (Fig. 14C), consistent with the results from TCGA and ICGC databases. The area under the ROC curve (AUC) for 1, 3, and 5 years were 0.826, 0.824, and 0.836, respectively (Fig. 14D). Therefore, all these findings consistently confirmed the predictive performance of the prognostic model constructed above, indicating its better credibility and effectiveness in predicting prognosis for SARC patients.

## Discussion

In disulfidptosis, an excessive accumulation of disulfide molecules inside cells leads to disulfide stress and subsequent cell death. Understanding the regulatory mechanism, signaling pathways, and pathological associations of disulfidptosis are currently limited. Mechanistic studies of disulfidptosis represent a novel and important direction in disease therapy. In the context of malignant tumors, intracellular disulfide-mediated cell death plays a role in oxidative stress within cells, and the formation of invasive pseudopods may lead to more disulfide stress in metastatic malignant tumor cells^16^. Specifically, the regulatory factors of disulfidptosis in sarcoma (SARC) remain poorly understood. To improve our understanding of the expression pattern, prognostic value, and potential mechanisms of Disulfide-Related Genes (DRGs) in SARC, we conducted a series of studies on the 24 existing DRGs. Our findings were further validated through RT-qPCR, providing valuable insights for future research on DRGs.

This study identified two subtypes of disulfidptosis associated with DRGs. We have observed the DRG involvement in various signaling pathways by comparing the DEGs between these subtypes. These included ECM-receptor interaction, focal adhesion, cGMP-PKG signaling pathway, cAMP signaling pathway, PI3K-Akt signaling pathway, Wnt signaling pathway, PD1 signaling, B cell receptor signaling pathway, collagen formation, glycosaminoglycan metabolism, and P53 downstream pathway. These pathways have been extensively discussed because of their close association with tumor invasion and metastasis. The extracellular matrix (ECM) is an essential factor in cancer progression. ECM receptor interaction pathway is a key pathway leading to the occurrence, progression, metastasis, and response to cancer treatment^17^. Guo et al.^18^ observed that ACSM1 significantly contributes to the carcinogenicity of prostate cancer, influencing metabolic pathways and ECM-receptor interaction signaling pathways. Focal adhesion is crucial in the invasiveness and metastasis of tumors. Zhang et al.^19^ observed the involvement of CPNE8 in regulating focal adhesion and TME to improve progression and invasiveness in gastric cancer, indicating its potential as a novel prognostic biomarker. Collagen is the principal structural protein and a vital constituent of the ECM, possessing paramount importance. Its impact on tumor progression has been demonstrated by regulating multiple cellular processes, such as cell proliferation, migration, invasion, and metastasis. Specifically, collagen can facilitate the growth and invasiveness of cancer cells while inhibiting programmed cell death (apoptosis) and extending their survival. Therefore, collagen is a potentially effective therapeutic target in the cancer treatment^20,21^. Li et al.^22^ reported that in an inflammatory microenvironment induced by lipopolysaccharide (LPS), STEAP4 silencing in prostate cancer cells activates the cGMP-PKG pathway, reducing cell proliferation. Cervical cancer cells indicate elevated levels of lncRNA DARS-AS1, leading to heightened cell proliferation, invasion, and migration by activating the cGMP-PKG pathway. Previous research suggests that the pathway is significantly associated with the overall survival^23^. The growth and metabolic activities of ovarian cancer are predominantly controlled by the cAMP-dependent pathway, which is crucial in tumor transformation, metastasis, proliferation, and apoptosis inhibition^24^. The PI3K/Akt signaling pathway is implicated in diverse biological processes and is indispensable for cellular proliferation and apoptosis, significantly advancing tumors. For example, ZIP10 stimulates the proliferation and chemoresistance of osteosarcoma cells by activating the PI3K/AKT pathway via ITGA10-mediated mechanisms^25^. Hawkins et al.^26^ first reported that tumor cells in Ewing sarcoma activate the Wnt/β-catenin signaling pathway, upregulating ECM protein secretion.

The constructed DRG prognosis model was closely associated with the clinical outcomes of SARC, followed by identifying three prognosis-related genes (*SLC7A11, RPN1, GYS1*). Several studies have depicted a strong association between these signature genes and tumors. Solute carrier family seven-member 11 (SLC7A11) has emerged as a promising biomarker and therapeutic target in cancer diagnosis and treatment. Additionally, the proposal of the disulfidptosis theory and the ongoing elucidation of regulatory factors provide novel avenues to investigate highly expressed disulfide bond proteins and glucose-dependent tumor diseases. Chen et al.^27^ have depicted that KDM4A exerts transcriptional control over SLC7A11 by modulating H3K9me3 demethylation in the promoter region of SLC7A11 and promoting osteosarcoma cell ferroptosis. In addition, Shi et al.^28^ reported that tirapazamine partially inhibits the proliferation and migration of osteosarcoma cells by inhibiting SLC7A11-induced ferroptosis. The Ribophorin 1 (RPN1) oligosaccharyltransferase (OST) complex is critical in N-linked glycosylation. Suppression of RPN1 expression hampers the proliferation and invasion of breast cancer cells while also inducing apoptosis via endoplasmic reticulum stress^29^. Additionally, Glycogen synthase 1 (GYS1) is the principal rate-limiting enzyme in the final stage of glycogen synthesis^30^. Chen et al.^31^ have elucidated that GYS1 triggers glycogen accumulation through the NF-κB pathway in clear cell renal cell carcinoma, fostering tumor progression. Consequently, the three prognostic DRGs identified in this study strongly correlate with malignant tumor invasion and progression, demanding additional investigation.

The identification of prognostic features and new prognostic biomarkers will aid in patient stratification and the development of individualized precision treatment strategies. Particularly, several gene expression-based prognostic features have been developed, some of which have been applied in clinical trials and practice^32^. For instance, Wang et al.^33^ developed a disulfide-related risk model to predict the prognosis and immune features of glioma patients, serving as an independent prognostic factor for glioma. Yue et al.^34^ constructed a prognostic model based on genes associated with programmed cell death, demonstrating high predictive accuracy for prognosis in melanoma patients. Prognostic features based on disulfide-related genes in lung adenocarcinoma effectively predict prognosis and are closely associated with the sensitivity to chemotherapy drugs and response to immunotherapy^35^. Cellular senescence of tumor cells plays a crucial role in tumor progression, and a robust nomogram constructed with senescence-related and clinical features can predict the survival rate of each patient^36^. Prognostic features related to copper death genes in hepatocellular carcinoma are utilized to predict the survival probability of HCC patients^37^. Additionally, Liu et al.^38^ identified four pseudo-gene features associated with survival in osteosarcoma through machine learning, effectively distinguishing high-risk from low-risk patients with high sensitivity and specificity for prognosis, further validated by ROC curves, demonstrating superior performance of the combined model in prognosis assessment.

The current study employed a LASSO Cox regression to construct a three-gene prediction model. The Kaplan–Meier curve demonstrated that patients classified as high-risk according to the model exhibited a significantly worse prognosis than those in the low-risk group. Moreover, the ROC curves for the probability of survival at 1, 3, and 5 years depicted that the prognostic model showed positive specificity and sensitivity. The effectiveness and stability in predicting the prognosis of SARC patients have been validated using TCGA internal and ICGC external validation cohorts. Finally, comparing the ROC curves of TCGA, ICGC, and the combined model, it was found that the combined model had better predictive performance than the other cohorts. However, their AUCs were also relatively close, indicating that the three models may have similar performance and require validation by a large-scale cohort. Univariate and multivariate Cox analysis helped confirm the model's status as an independent prognostic factor for SARC. Additionally, a predictive nomogram was developed with the signatures to forecast the clinical outcomes of SARC patients.

Evidence indicates that the infiltration of immune cells is crucial in the development and spread of SARC^39,40^. This study revealed a significant association between the prognostic DRGs and the abundance of specific immune cells, with elevated infiltration abundance associated with improved clinical prognosis for SARC. Furthermore, prognostic DRGs exhibited strong connections to TAMs and CAFs-related markers while displaying a significant positive correlation with EMT-related markers. Tumor-associated macrophages (TAMs) develop an important TME constituent, exerting crucial functions in tumor initiation and facilitating malignant progression, encompassing tumor angiogenesis and metastasis^41^. Within the macrophage population, M2-like macrophages prevail, and their abundance indicates an immunosuppressive milieu in the tumor. Cersosimo et al.^42^ have provided evidence that the infiltration of M2-like TAMs correlates with heightened metastatic potential and unfavorable prognosis among osteosarcoma patients. However, the present study has depicted a positive correlation between increased levels of macrophages M1 and M2 cells in sarcomas with favorable prognosis. Additional evidence and discourse may help elucidate the precise mechanisms via which DRGs and immune cell phenotypes affect the prognosis of sarcoma patients.

Cancer-associated fibroblasts (CAFs) represent the predominant cellular constituents in TME, and their involvement in cancer progression is manifested by facilitating cancer stem cell renewal, immune therapy resistance, and chemical agent resistance^43,44^. Moreover, CAFs significantly promote tumor heterogeneity by involving ECM protein deposition and dynamic TME remodeling^45^. This finding aligns with the GSEA results mentioned earlier. Huang et al.^46^ discovered that CAFs exhibit heightened invasiveness in recurrent osteosarcoma and are enriched in the epithelial-mesenchymal transition (EMT) pathway. The researchers examined the association between DRGs and EMT-related markers to explore the impact of DRGs on EMT in sarcomas. The study revealed a positive association between DRGs and EMT-related markers. Hence, there is a requirement for additional investigation into the immunosuppressive characteristics of CAFs in SARC. Although existing studies have primarily evaluated cellular components, the precise role and TME mechanism in SARC progression remain unclear. This study employed scRNA-seq and bioinformatics techniques to uncover the regulatory function of CAFs in TME of SARC. The findings indicate that the dysregulated expression of DRGs in SARC may remodel the TME, affecting invasion and metastasis. Despite the novel study findings, it is imperative to acknowledge the existing limitations, necessitating additional exploration. Further investigation is warranted to demonstrate potential mechanisms and conduct clinical trials, providing fresh perspectives on managing primary and recurrent SARC patients.

Recently, immune checkpoint inhibitors (ICIs) have revolutionized cancer treatment and expanded their application through various indications. Identifying immune checkpoints, including PD-1, PD-L1, and CTLA-4, is pivotal in advancing cancer immunotherapy^47,48^. The human leukocyte antigen (HLA) molecule is a conduit for the anti-tumor immune response with a critical function^49,50^. This study investigated the correlation between the expression of DRGs and immune checkpoint genes and HLA members. Our analysis revealed that CTLA4, HAVCR2, TIGIT, and PDCD1 indicated elevated expression levels in group C1. Moreover, certain HLA members demonstrated significantly higher expression in group C1 than in group C2. This aligned with the expression of HLA members and the infiltration of antigen-presenting cells observed in this study. Furthermore, the TIDE score was lower in group C2, indicating that such patients may benefit from ICI treatment. The findings strongly indicate an association between DRGs and the immune response to SARC, indicating the potential utilization of DRGs as an immunotherapy target in SARC. Nevertheless, it is imperative to investigate and validate predictive biomarkers in clinical cohorts to improve the clinical outcomes of SARC patients.

Previous studies have reported that feature models related to Disulfide-Related Genes (DRGs) can predict patients' response to immunotherapy, but there is almost no research on DRGs in SARC patients, and most studies have not been validated in external independent datasets and clinical samples^51–53^. We further collected clinical tissue samples from 24 patients at our hospital, Chaohu Hospital of Anhui Medical University, to validate the expression levels of genes in the patients' DRGs prognostic model and their prognosis. After dividing patients into high-risk and low-risk groups, we found a significant enrichment of patients with poor clinical prognosis in the high-risk group. Additionally, we collected two external independent immunotherapy cohorts (anti-PD-1/PD-L1/CTLA-4) and a cohort of late-stage sarcoma patients who received immunotherapy at our hospital to evaluate the performance of DRGs in predicting immunotherapy response. The results showed that DRGs have good predictive ability for patient immune response, and patients in the high-risk group are more suitable for immunotherapy.

In some SARC patients, immunocheckpoint inhibition has demonstrated therapeutic efficacy in clinical contexts. However, the response rate to ICIs remains limited to a small subset of patients, necessitating the identification of predictive biomarkers to identify individuals more likely to benefit from immunotherapy. TMB and MSI have emerged as potential predictive biomarkers for the ICI response^54,55^. MSI contributes to the accumulation of somatic mutations, thereby leading to enhanced tumor mutation burden, novel antigen generation, and the activation of immune effector cells and anti-tumor immune response^56,57^. Furthermore, evidence indicates a substantial association between cancer cell stemness and their ability to evade and resist the immune system^58^. Based on our previous demonstration of the correlation between DRGs and prognosis and immune cell infiltration in SARC patients, we evaluated the relationship between DRG risk scores and TMB, MSI, and mRNAsi. Our study indicated a significant positive correlation between risk scores and the TMB, MSI, and mRNAsi scores of high-risk groups, significantly higher than low-risk groups. Moreover, SARC patients exhibiting high TMB, MSI, and mRNAsi scores experienced a shorter overall survival. Therefore, the presence of high TMB, MSI, and mRNAsi groups indicates an unfavorable prognosis and heightened likelihood of disease progression among SARC patients. Additionally, DRG prognosis is influenced either positively or negatively by different chemotherapy and targeted drugs. However, further experiments are necessary to validate this association. These findings present novel therapeutic targets for treating SARC.

DNA methylation is an epigenetic process governing the regulation of gene expression^59^. Alterations in DNA methylation patterns are valuable biomarkers for the timely identification, diagnosis, prediction of outcomes, and assessment of treatment response^60–63^. Győrffy et al.^64^ demonstrated the effect of aberrant DNA methylation on gene expression and prognostic outcomes in different breast cancer subtypes. Notably, abnormal DNA methylation predominantly manifests during the initial stages of malignant tumor development. Li et al.^65^ provided a comprehensive overview of the application of liquid biopsy using DNA methylation for early detection and prognosis assessment of lung cancer. Moreover, the authors elucidated that liquid biopsy based on DNA methylation can identify and monitor primary and metastatic malignant tumors while assessing tumor heterogeneity. Qin et al.^66^ demonstrated that m7G-related genes exhibit significantly upregulated expression in SARC and established an m7G-related prognostic model for predicting the survival rate of sarcoma patients. Presently, there exists a dearth of research investigating the association between DNA methylation and early diagnosis and prognosis in sarcoma patients, along with the potential of DNA methylation analysis to become a predictive indicator of sarcoma treatment efficacy. Prior research has demonstrated that elevated levels of DNA methylation in promoters impede the process of gene transcription. Conversely, decreased levels of DNA methylation in promoters facilitate gene transcription. The excessive methylation of tumor suppressor genes or the insufficient methylation of oncogenes depict a strong association with the advancement and progression of malignant tumors^67^. Our study established that the methylation status of DRGs is negatively correlated with SARC. The study also revealed a positive correlation between the methylation of DRGs and the expression of most genes involved in RNA epigenetic modification. This helps establish a connection with unfavorable prognosis among SARC patients. Therefore, the methylation status of DRGs can be a potential prognostic indicator for early detection and prognosis assessment in SARC patients.

Another significant study finding involves the investigation of the SARC-related regulatory axis comprising SLC7A11/hsa-miR-29c-3p/LINC00511, RPN1/hsa-miR-143-3p/LINC00511. This potentially contributes to the invasion and metastasis of SARC. The aberrant expression of hsa-miR-29c-3p and hsa-miR-143-3p is crucial in various tumorigenic processes. Previous studies have documented the downregulation of hsa-miR-29c-3p within head and neck squamous cell carcinoma, associated with higher tumor grade and unfavorable overall survival and disease-specific survival outcomes^68^. Downregulation of hsa-miR-29c-3p is an independent prognostic factor for laryngeal squamous cell carcinoma (LSCC) and is linked with poorer outcomes^69^. Ju et al.^70^ described that the expression of has-miR-143-3p was significantly downregulated in gastric cancer, potentially becoming a diagnostic biomarker. Circular RNA circCSNK1G3 can induce HOXA10 expression via the sponge of has-miR-143-3p, improving the growth and metastasis of lung adenocarcinoma cells^71^. Evidence indicates that the disruption of lncRNA expression is significant in developing numerous cancers associated with cell growth and programmed cell death^72^. Furthermore, long non-coding RNAs (lncRNAs) play a crucial role in regulating various biological functions and have been found to play a key role in immune regulation^73^. Nevertheless, the precise biological role and governing LINC00511 mechanism in SARC remain largely uninvestigated. Liu et al.^74^ indicate that DNA hypomethylation enhances the expression of LINC00511 in breast cancer. Thus, elevated expression of LINC00511 is closely linked with clinical pathological features and poor prognosis in breast cancer patients. Furthermore, LINC00511 overexpression enhances the proliferation of gastric cancer cells by functioning as a competing endogenous RNA (ceRNA) to regulate the miR-124-3p/PDK4 pathway^75^.

## Conclusion

In summary, we have successfully constructed a prognostic risk model for SARC comprised of three prognostic-related DRGs (*SLC7A11, RPN1, GYS1*). We have identified significant correlations between the expression of DRGs and clinical prognosis of SARC patients, tumor immune cell infiltration levels, DNA methylation, immune therapy response, and drug sensitivity. Furthermore, we have identified relevant regulatory axes that may play important roles in the development of sarcomas. Additionally, we have further validated the performance of DRG risk score features in predicting survival outcomes of SARC patients and determining sensitivity to immune checkpoint inhibitor (ICI) therapy using clinical tissue sample data and external datasets. We have reason to believe that our research may provide valuable insights for clinical decision-making and personalized treatment strategies, and holds promise as potential prognostic biomarkers, therapeutic targets, and predictive factors for immune therapy response in SARC patients.

## Materials and methods

### Data sources and preprocessing

This study is based on The Cancer Genome Atlas (TCGA) (https://portal.gdc.cancer.gov//)^76^ RNAseq data and corresponding clinical information for SARC were retrieved from the TCGA database. The study involved 260 SARC patients and two normal tissue samples. The data used in the study were standardized data per million transcripts (Transcripts Per Million, TPM). Moreover, the data distribution was close to normal, ensured by the R software (v4.0.3) “ggplot2”. The gene expression data were extracted to build a data matrix and analyzed using the Wilcoxon test.

### Establishment of subtypes

According to previous literature, we identified 24 potential disulfidptosis-related genes (DRGs)^5^ (Supplementary Table S1). Based on the consistent clustering of the 24 genes, the R software package ConsensusClusterPlus (v1.54.0)^77^ was used for consistency analysis. The maximum number of clusters is 6 (k = 6), and 80% of the total sample is drawn 100 times, clustering = “hc”, innerLinkage = ‘ward.D2’. The number of clusters varies from 2 to 6 (k = 2–6), and the consistency matrix and the consistency cumulative distribution function (CDF) were assessed together to determine the best classification. The R software package pheatmap (v1.0.12) analyzed the clustering heat maps. The gene expression heat maps retained the motifs using a variance above 0.1. TCGA cases were divided into Cluster1 (C1) and Cluster2 (C2) based on the DRG expression profiles.

### Identification and enrichment analysis of differentially expressed genes

DEGs between C1 and C2 subtypes were identified using the Limma package (v3.40.2) in R software^78^. The adjusted *p* value was analyzed within the TCGA database to correct for false positives. “Adjusted *p* < 0.05 and log2 (Fold change) > 1.5 or log2 (Fold change) <  − 1.5” is characterized as the standard for screening differential expression of mRNA. R software heatmap package helped draw the heat map. The Gene Ontology (GO) function of DEGs and its enrichment in the Kyoto Encyclopedia of Genes and Genomes (KEGG) pathway were assessed using the R package “clusterProfiler” (v3.18.0)^79^. Moreover, gene set enrichment analysis (GSEA) (http://software.broadinstitute.org/gsea/index.jsp)^80^ helped identify potential biological pathways. Based on TCGA data, DEGs were divided into up-regulated and down-regulated groups. Each analysis tested 10,000 gene combinations to identify pathways with significant changes. The gene was enriched to meaningful pathways when p.adjust < 0.05 and FDR (false discovery rate) < 0.25. GeneMANIA (http://www.genemania.org)^81^ helps elucidate the relationship between genes and datasets by constructing gene interaction networks. This software helped visualize the DRG network in terms of physical interaction, co-expression, prediction, co-mapping, and genetic interaction and evaluate the DRG network function.

### Genetic variation

Gene Set Cancer Analysis (GSCA) (http://bioinfo.life.hust.edu.cn/GSCA/#/)^82^ helped integrate expression, mutation, drug sensitivity, and clinical data from four public data sources for 33 cancer types. The somatic mutations of SARC 260 patients were retrieved and visualized using the maftools package in R software, involving four mutation types: Missense_Mutation, Nonsense_Mutation, Splice_Site, and Multi_Hit. This study also analyzed the Spearman correlation between the expression of DRG mRNA and CNV methylation. We investigated the correlation between methylation, CNV, and survival outcomes in SARC patients. This included Disease Free Interval (DFI), Disease Specific Survival (DSS), Overall Survival (OS), and Progression Free Survival (PFS).

### Relationship between DRGs and clinicopathological features and prognosis in SARC

The Kaplan–Meier curve, *p* value, and hazard ratios (HRs) with 95% Confidence intervals (CIs) were obtained through log-rank test and univariate Cox regression analysis. Subsequently, some essential prognostic DRGs (*SLC7A11, RPN1,* and *GYS1*) of SARC patients were screened and analyzed. The relationship between prognosis-related DRGs and the overall survival rate of SARC patients was analyzed, and the area under the receiver operating characteristic curve (ROC) was calculated. TARGET (https://ocg.cancer.gov/programs/target)^83^ is an open database for pediatric cancer, and we obtained 89 osteosarcoma cases from the Target database. The expression differences between high and low DRGs expressing groups in SARC were validated through 34 osteosarcoma cases in the GSE16091 dataset and 310 SARC cases in the GSE21050 dataset obtained from the NCBI-GEO database (https://www.ncbi.nlm.nih.gov/gds)^84^.

In addition, we compared the prognostic outcomes between the high and low-risk groups of three prognostic DRGs in clinical pathological features. We obtained the corresponding clinicopathological data of 260 SARC patients from TCGA, including Age, Sex, Race, New Tumor, Radiation, Neoadjuvant, and Therapy.

### Construction and validation of the DRG prognostic model

According to the above DRG levels associated with SARC prognosis, LASSO-Cox regression analysis helped present the prognostic model. After conducting tenfold cross-validation, we obtained the minimum value of the tuning parameter lambda. We selected this minimum lambda value because it performed the best on the validation dataset. Subsequently, we used this optimal lambda value to fit the LASSO Cox regression model. Cross-validation is a widely accepted method for assessing the generalization ability of predictive models by repeatedly training and evaluating the model on different subsets of the data. By selecting lambda based on this rigorous cross-validation process, we ensured the robustness and reliability of the model selection process. The multivariate Cox regression analysis helped measure the prognostic DRGs risk score: Riskscore = ∑_i_ Coefficient (mRNA_i_) × Expression (mRNA_i_). Subsequently, the whole TCGA-SARC (n = 260) dataset was utilized as the training cohort. According to the average risk score, TCGA-SARC patients were divided into low-risk and high-risk subtypes. The overall survival rates of the two subtypes were compared using the Kaplan–Meier analysis, and time ROC analysis helped predict the model’s accuracy. The optimal truncated expression value is determined using the “surve_cutpoint” function of the “surviver” R package. Then, the validation cohort helped verify the accuracy of the DRG signature using the above formula; SARC patients in the TCGA SARC dataset were randomly divided into validation set 1 (n = 130) and validation set 2 (n = 130). Moreover, the ICGC (http://dcc.icgc.org) dataset became the external validation cohort, verifying the above result.

### Building a predictive nomogram

Forest plots helped display each variable (including *p* values, HR, and 95% CI) through univariate and multivariate Cox regression analysis using the "forestplot" package. The “rms” package helped build a Nomogram model for predicting 1-, 3-, and 5-year overall survival (OS) and disease-specific survival (DSS) according to the results of multivariate Cox proportional hazards analysis.

### Analysis of gene expression related to immune infiltration and immune checkpoints

For immune scoring, the R software immunedeconv package^85^ and six most advanced algorithms, involving TIMER^86^, xCell^87^, MCP-counter^88^, CIBERSORT^89^, EPIC^90^, and quantTIseq^91^, helped compare the degree of immune cell infiltration between C1 (N = 187) and C2 (N = 73) subtypes using the Wilcoxon test. Gene Expression Profiling Interactive Analysis (GEPIA) (http://gepia.cancer-pku.cn/index.html)^92^ database has explored the association between immune cell infiltration and prognosis of SARC patients. Moreover, the single-sample gene set enrichment analysis (ssGSEA) in R packet “GSVA”^93^ helped quantify the infiltration level of immune cell types and the infiltration and accumulation of 23 common immune cells, such as dendritic cells (DC), immature DC (iDC), activated DC (aDC), cytoplasmic DC (pDC), T helper (Th) cells, type 1 Th cells (Th1), type 2 Th cells (Th2), type 17 Th cells (Th17), regulatory T cells (Treg), T gamma delta (Tgd), T central memory (Tcm), T effect memory (Tem), T follicular helper (Tfh), CD8 + T cells, B cells, neutrophils, macrophages, cytotoxic cells, mast cells, eosinophils, natural killer (NK) cells, NK56-cells, and NK56 + cells. Wilcoxon rank sum test helped compare the differences in immune cell infiltration levels of three prognosis-associated DRG levels between high and low expression groups. Finally, the Spearman correlation helped explore the relationship between three prognosis-linked DRGs and immune cell infiltration.

The expression levels of some immune checkpoint-related genes (*CD274, CTLA4, HAVCR2, LAG3, PDCD1, PDCD1LG2, TIGIT,* and *SIGLEC15*), and the human leukocyte antigen (HLA) members (*HLA-A, HLA-B, HLA-C, HLA-DMA, HLA-DMB, HLA-DOA, HLA-DOB, HLA-DPA1, HLA-DPB1, HLA-DQA1, HLA-DQA2, HLA-DQB1, HLA-DRA, HLA-DRB1, HLA-E, HLA-F, HLA-G, TAP1,* and *TAPBP*) were analyzed. The Tumor Immune Dysfunction and Exclusion (TIDE) algorithm^94^ predicted the potential immune checkpoint-blocking response, while the results were visualized with R (v4.0.3) packages “ggplot2” and “pheatmap”.

### TMB, MSI, ESTIMATE, mRNAsi, and drug sensitivity analysis

The association of three DRGs in SARC with tumor mutation burden (TMB), microsatellite instability (MSI), the Estimation of Stromal and Immune cells in MAlignant Tumor tissues using Expression data (ESTIMATE), and mRNA stemness index (mRNAsi) was analyzed using Spearman correlation. The sensitivity of these drugs, followed by an understanding of the correlation between the critical immune checkpoints and the four DRG levels, were studied. Immune cell abundance (immune score), stromal cell infiltration level (stromal score), and tumor purity (ESTIMATE score) were estimated with the ESTIMATE algorithm. The OCLR algorithm helped calculate mRNAsi, which was constructed by Malta et al.^95^ Based on the mRNA expression signature, the gene expression profile contains 11,774 genes. We utilized the same Spearman correlation (RNA expression data). The minimum value was subtracted, and the result was divided using the maximum maps of the dryness index to the range [0,1]. The chemotherapy response for each sample was predicted using the Genomics of Drug Sensitivity in Cancer (GDSC) (https://www.cancerrxgene.org/)^96^ and the Cancer Therapeutics Response Portal (CTRP) (https://portals.broadinstitute.org/ctrp/) database. The 50% inhibiting concentration (IC50) of chemotherapeutic drugs was predicted using the R package pRRophetic^97^, in which the IC50 value of the sample was estimated through ridge regression. All the parameters were set according to the default value. The batch effect of combat and the tissue type were all used. Moreover, the duplicate gene expression was summarized as the mean value. This study integrated the drug sensitivity and gene expression profile data from cancer cell lines in the GDSC using the CTRP database.

### Single-cell analysis

The expression of DRGs in the TME was analyzed using the Tumor Immune Single Cell Center (TISCH) (http://tisch.comp-genomics.org/)^98^ based on the prognosis of SARC patients. TISCH is a single-cell RNA sequencing (scRNA-seq) database focused on TME. TISCH provides extensive annotations of cell types at the single-cell level, helping explore the TME of different cancer types. This dataset comprises three main cell types: immune, stromal, and malignant. In this study, the t-distributed stochastic neighborhood embedding (t-SNE) map of SARC_GSE119352_aPD1aCTLA4 and the heat map of SARC_GSE119352_aPD1aCTLA4 were represented through the TISCH database to indicate the effect of DRGs on the TME in SARC. Simultaneously, the scatter diagram of the correlation between the immune infiltration level of DRGs and cancer-associated fibroblasts (CAFs) and macrophages was drawn using TIMER2.0 (http://timer.cistrome.org/)^99^.

### Relationship between DRG expression level and RNA modification regulatory factors

We analyzed the differences in gene expression between high and low-risk groups for m6A, m5C, m1A, and m7G genes in 260 SARC samples and the correlation between the prognostic DRGs in SARC samples and m6A, m5C, m1A, and m7G genes expression using the Wilcoxon test and the "ggplot2" package in R software (v4.0.3). The expression matrix for m6A gene involves (*RBM15B, VIRMA, IGF2BP2, HNRNPA2B1, IGF2BP1, YTHDF3, IGF2BP3, HNRNPC, RBM15, RBMX, METTL14, YTHDC2, METTL3, ZC3H13, WTAP, YTHDF1, YTHDC1, FTO, YTHDF2,* and *ALKBH5*). m5C gene includes (*DNMT1, DNMT3A, DNMT3B, MBD1, MBD2, MBD4, MECP2, TDG, UHRF1, UHRF2, UNG, ZBTB33, ZBTB38, ZBTB4, TET1, TET2, TET3*); m1A gene includes (*TRMT10C, TRMT61B, TRMT6, ALKBH3, ALKBH1, YTHDC1, YTHDF1, YTHDF2, YTHDF3*); m7G gene includes (*AGO2, CYFIP1, DCP2, EIF4A1, EIF4E, EIF4G3, GEMIN5, LARP1, NCBP1, NCBP2, NCBP3, NSUN2, NUDT11, NUDT16, NUDT4, SNUPN,* and *WDR4*).

### Prediction of potential microRNA and long non-coding RNA target genes

RNA22 (https://cm.jefferson.edu/rna22/interactive)^100^, TargetMiner (https://www.isical.ac.in/~bioinfo_miu/targetminer20.htm)^101^, and miRWalk (http://miRWalk.umm.uni-heidelberg.de/)^102^ databases helped screen candidate microRNA (miRNA) and predict miRNA targets. These selected miRNAs are called miRNAs of target genes. The possible combination of long non-coding RNAs (lncRNAs) and miRNAs was predicted using miRNet (http://www.mirnet.ca/)^103^ and ENCORI (http://starbase.sysu.edu.cn/)^104^ database. Subsequently, the mRNA-miRNA and miRNA-lncRNA regulation networks were established using Cytoscape (version 3.7.1; http://www.cytoscape.org/)^105^. In addition, the correlation and prognostic value of these candidate miRNAs and lncRNAs in SARC were verified using ENCORI and the Kaplan–Meier plotter database.

### Human specimens

The Chaohu Hospital of Anhui Medical University provided all tissue samples for this study, including 24 pairs of sarcoma tissues and adjacent tissues, along with follow-up formation for all patients. The tissue specimens were embedded in 10% formalin. All patients underwent pathological examination for definitive diagnosis. The use of sarcoma samples in this study was approved by the Ethics Committee of Chaohu Hospital of Anhui Medical University (Approval No: KYXM202310004), and informed consent was obtained from all patients. All experiments were performed in accordance with relevant guidelines and regulations.

### Cell culture

Cell lines, including 143B, SW982, SW872, osteoblast cell line (hFOB1.19, Punosai, Wuhan, China), synovial fibroblast (HFLS, Jennio Biotech, Guangzhou, China), and human preadipocyte line (HPA-v, sciencell) were utilized in this study. All the cell lines were cultured in Dulbecco modified Eagle medium (DMEM; Gibco, Grand Island, NY, United States) supplemented with 10% fetal bovine serum (Gibco, Grand Island, NY, United States), 100 U/ml penicillin and 100 U/ml streptomycin (Invitrogen, Carlsbad, CA, United States). The hFOB1.19 cell line was cultured within an incubator containing 5% CO_2_ at 34 °C, while the remaining cells were cultured inside a 5% CO_2_ incubator at 37 °C.

### RNA isolation and RT-qPCR

Total RNA was extracted from cultured cells using high-purity RNA separation kits (Roche Diagnostics, Mannheim, Germany) and DNase I (Roche Diagnostics, Sigma-Aldrich) based on the manufacturer’s instructions. RNA was directly reverse transcribed with HiScript ^®^II 1st Strand cDNA Synthesis Kit (MR101-01 MR101 V azyme, Nanjing, China) based on the manufacturer’s instructions. Then, AceTaq ^®^qPCR SYBR Green Master Mix (Q121-03 azyme V azyme, China) was utilized for quantitative RT-PCR. The amplification conditions were: pre-denaturation at 95℃ for 30 s, denaturation at 95℃ for 5 s, annealing at 60℃ for 30 s, and 40 cycles based on 1 μmol primer, 10 ng sample, 0.08 μmol ROX dye, and 2 × SYBR Green Pro TaqHS Premix II hybrid setting 20 μl reaction system. Several specific primer sequences (Gene Pharma, China) were designed, and the primer sequences are listed in Table 1. The mean cycle threshold (Ct) value of each target gene was normalized to the housekeeping gene GAPDH for PCR analysis. The results are represented in a fold change with the ^ΔΔ^Ct method.

**Table 1:** Primer sequences of genes.

Real-time quantitative PCR primer sequence	
Gene	Sequence (5′–3′ on minus strand)
GAPDH	Fwd: GGAGCGAGATCCCTCCAAAAT
	Rev: GGCTGTTGTCATACTTCTCATGG
SLC7A11	Fwd: TCTCCAAAGGAGGTTACCTGC
	Rev: AGACTCCCCTCAGTAAAGTGAC
RPN1	Fwd: GGCCAAGATTTCAGTCATTGTGG
	Rev: CTTCGTTGGATAGGGAGAGTAGA
GYS1	Fwd: GCGCTCACGTCTTCACTACTG
	Rev: TCCAGATGCCCATAAAAATGGC

### Statistical analysis

All statistical analyses were based on R (v4.2.1) (https://www.r-project.org/). The Wilcoxon rank sum test was used to compare differences between nonparametric data from two independent samples. Spearman or Pearson correlation analysis was used to calculate the correlation between the two groups. Kaplan–Meier survival analysis, univariate and multivariate Cox regression analysis were used to analyze the prognosis. In all analyses, *p* values < 0.05 were considered statistically significant (*p < 0.05, **p < 0.01, ***p < 0.001). Each section in this study was analyzed with specific datasets, R packages, and databases.

### Supplementary Information

Supplementary Information.

## Data Availability

The datasets are available in TCGA database (https://portal.gdc.cancer.gov/), NCBI-GEO (https://www.ncbi.nlm.nih.gov/gds), ICGC (http://dcc.icgc.org), GSCA (http://bioinfo.life.hust.edu.cn/GSCA/#/), GeneMANIA (http://www.genemania.org), GSEA (http://software.broadinstitute.org/gsea/index.jsp), GDSC database (https://www.cancerrxgene.org/), Therapeutics Response Portal (CTRP) (https://portals.broadinstitute.org/ctrp/), TISCH database (http://tisch.comp-genomics.org/), TIMER (https://cistrome.shinyapps.io/timer/), RNA22 database (https://cm.jefferson.edu/rna22/interactive), TargetMiner (https://www.isical.ac.in/~bioinfo_miu/targetminer20.htm), miRWalk (http://miRWalk.umm.uni-heidelberg.de/), ENCORI database (http://starbase.sysu.edu.cn/), RNAInter database (http://www.rnainter.org/), as well as miRNet database (http://www.mirnet.ca/).
