# Supplementary material for: Correlation analysis of disulfidptosis-related gene signatures with clinical prognosis and immunotherapy response in sarcoma
Source: Sci Rep. 2024 Mar 26;14:7158. doi: 10.1038/s41598-024-57594-x (PMC10966107; doi:10.1038/s41598-024-57594-x)
Supplement: Supplementary file 1 — Supplementary Information. [file 41598_2024_57594_MOESM1_ESM.pdf]

SUPPLEMENTAL DATA

Correlation Analysis of Disulfidptosis-Related Gene Signatures with Clinical Prognosis and Immunotherapy response in Sarcoma

Juan Xu [1], kangwen Guo [2], Xiaoan Sheng [1], Yuting Huang [1], Xuewei Wang [1], Juanjuan Dong [1]*, Haotian Qin [3,4*], Chao Wang [1*]

[1] Department of Oncology, Chaohu Hospital of Anhui Medical University, Hefei, China.

[2] Affiliated Hospital of Guangdong Medical University, Zhanjiang, China.

[3]National and Local Joint Engineering Research Center of Orthopaedic Biomaterials, Peking University, Shenzhen Hospital, Shenzhen, China.

[4]Department of Bone and Joint Surgery, Peking University Shenzhen Hospital, Shenzhen, China.

* Correspondence:

Chao Wang (wangchao10107@163.com)

Haotian Qin(qinht10001@163.com)

Juanjuan Dong, (xj2020092020@163.com)

Supplementary Table 1. The abbreviations of DRGs in this study.

DRGs Abbreviation	Full name
SLC7A11	solute carrier family 7 member 11
SLC3A2	solute carrier family 3 member 2
RPN1	ribophorin I
NCKAP1	NCK-associated protein 1
NUBPL	nucleotide binding protein-like
NDUFA11	NADH dehydrogenase (ubiquinone) 1 alpha subcomplex, 11, 14.7kDa
LRPPRC	leucine-rich pentatricopeptide repeat containing
OXSM	3-oxoacyl-ACP synthase, mitochondrial

NDUFS1	NADH dehydrogenase (ubiquinone) Fe-S protein 1, 75kDa (NADH-coenzyme Q reductase)
GYS1	glycogen synthase 1 (muscle)
ACTN4	actinin, alpha 4
ACTB	actin, beta
CD2AP	CD2-associated protein
CAPZB	capping protein (actin filament) muscle Z-line, beta
DSTN	destrin (actin depolymerizing factor)
FLNA	filamin A, alpha
FLNB	filamin B, beta
INF2	inverted formin, FH2 and WH2 domain containing
IQGAP1	IQ motif containing GTPase activating protein 1
MYH10	myosin, heavy chain 10, non-muscle
MYL6	myosin, light chain 6, alkali, smooth muscle and non-muscle
MYH9	myosin, heavy chain 9, non-muscle
PDLIM1	PDZ and LIM domain 1
TLN1	talin 1

Supplementary Table 2.Gene sets enriched in phenotype high.

ID	NES	p.adjust	FDR
REACTOME_PD_1_SIGNALING	2.224624	6.35e-05	0.0034
KEGG_NATURAL_KILLER_CELL_MEDIATED_CYTOTOXICITY	2.109234	7.11e-05	0.0034
KEGG_INTESTINAL_IMMUNE_NETWORK_FOR_IGA_PRODUCTION	2.096748	8.08e-05	0.0035
REACTOME_COLLAGEN_FORMATION	1.987445	0.0001	0.0041
KEGG_CYTOKINE_CYTOKINE_RECEPTOR_INTERACTION	2.176196	0.0002	0.0043
NABA_ECM_GLYCOPROTEINS	1.626452	0.0002	0.0053

REACTOME_GLYCOSAMINOGLYCAN_METABOLISM	1.733586	0.0002	0.0054
PID_P53_DOWNSTREAM_PATHWAY	1.655477	0.0003	0.0071
KEGG_WNT_SIGNALING_PATHWAY	1.591576	0.0005	0.0096
KEGG_TOLL_LIKE_RECEPTOR_SIGNALING_PATHWAY	1.608973	0.0018	0.0260

Supplementary Table 3. The clinical characters of SARC patients in TCGA cohort

Clinical characters	Number
Alive	161
Dead	99
Mean (SD)	60.6 (14.7)
FEMALE	141
MALE	119
ASIAN	6
BLACK	18
WHITE	227
Metastasis	68
Primary	8
Recurrence	49
Non-radiation	140
Radiation	74
Neoadjuvant	1
No neoadjuvant	259
Ancillary:Chemotherapy	1
Ancillary:Chemotherapy:Hormone Therapy	1
Chemotherapy	59
Chemotherapy:	1

Chemotherapy:Hormone Therapy	2
Chemotherapy:Immunotherapy	3
Chemotherapy:Immunotherapy:Targeted Molecular therapy	1
Chemotherapy:Targeted Molecular therapy	3
Hormone Therapy	1

Supplementary Table 4. Correlation analysis between lncRNA and hsa-miR-29c-3p , hsa-miR-143-3p or lncRNA and SLC7A11, RPN1 in SARC determined by ENCORI databases.

LncRNA	miRNA	R value	p value
EBLN3P	hsa-miR-29c-3p	-0.127	4.08E-02
LINC00943	hsa-miR-29c-3p	-0.166	7.14E-03
LINC00511	hsa-miR-29c-3p	-0.354	3.90E-09
LINC01806	hsa-miR-143-3p	-0.126	4.21E-02
LINC01554	hsa-miR-143-3p	-0.247	5.35E-05
LINC00944	hsa-miR-143-3p	-0.440	8.69E-14
LINC00511	hsa-miR-143-3p	-0.303	6.17E-07
SMIM25	hsa-miR-143-3p	-0.268	1.10E-05
MIR503HG	hsa-miR-143-3p	-0.375	4.06E-10
**LncRNA**	**mRNA**	**R value**	**p value**
EBLN3P	SLC7A11	0.171	5.34E-03
LINC00943	SLC7A11	0.162	8.61E-03
LINC00511	SLC7A11	0.293	1.28E-06
LINC01806	RPN1	0.178	3.74E-03
LINC01554	RPN1	0.131	3.34E-02
LINC00944	RPN1	0.281	3.82E-06
LINC00511	RPN1	0.182	3.00E-03
SMIM25	RPN1	0.444	3.68E-14
MIR503HG	RPN1	0.201	1.06E-03

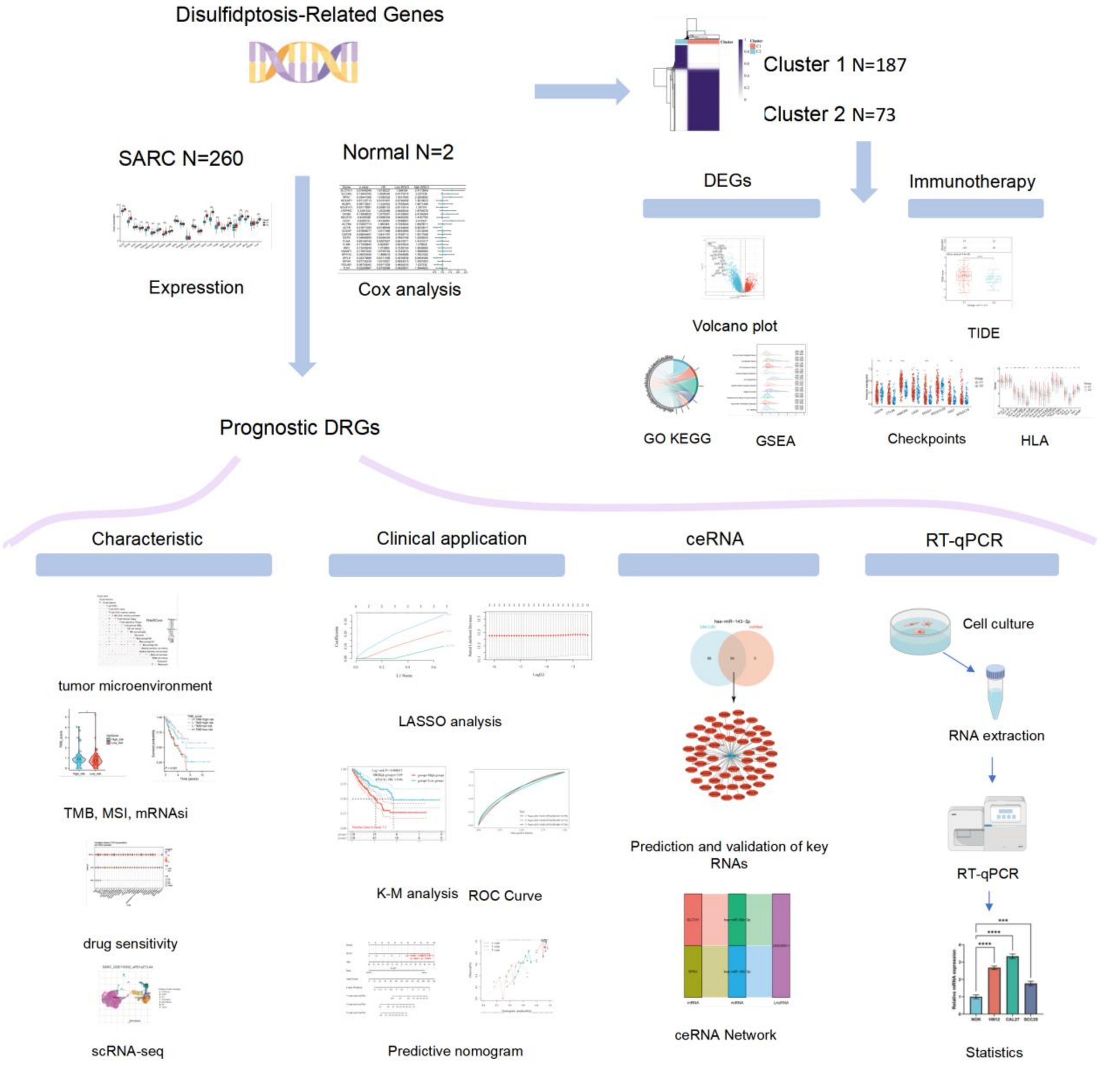

Fig. S1. Flowchart of the present study.

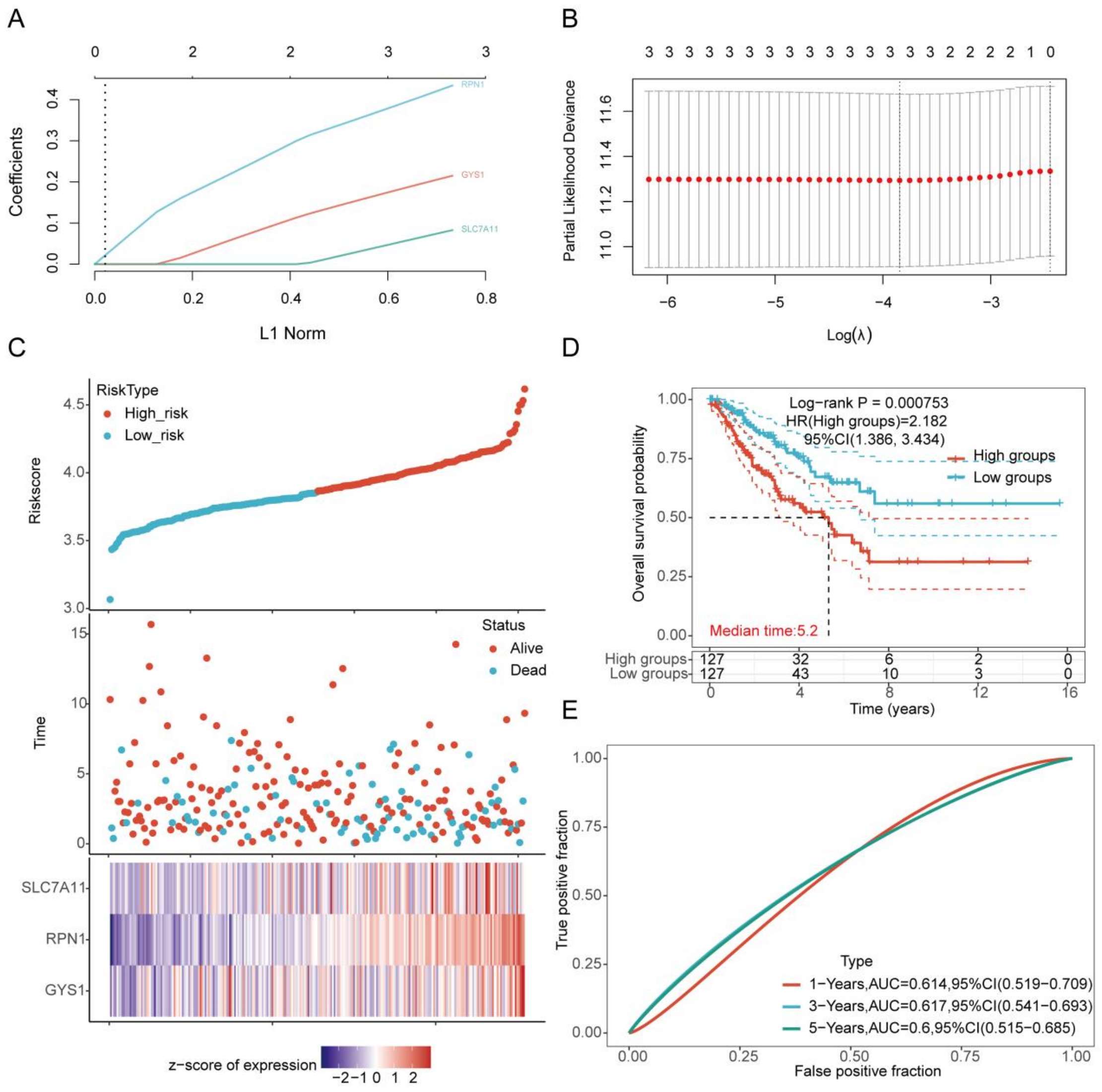

Fig. S2. Construction of a DSS model for SARC prognosis with the help of DRG. (A) LASSO coefficient curve of three DRGs; (B) Plots of the ten-fold cross-validation error rates; (C) Distribution of risk score, survival status, and expression of prognostic DRGs in SARC patients; (D) DSS curve of SARC patients in high/low-risk groups; (E) Time-dependent ROC curve for 1-, 3-, and 5-year DSS for DRGs.

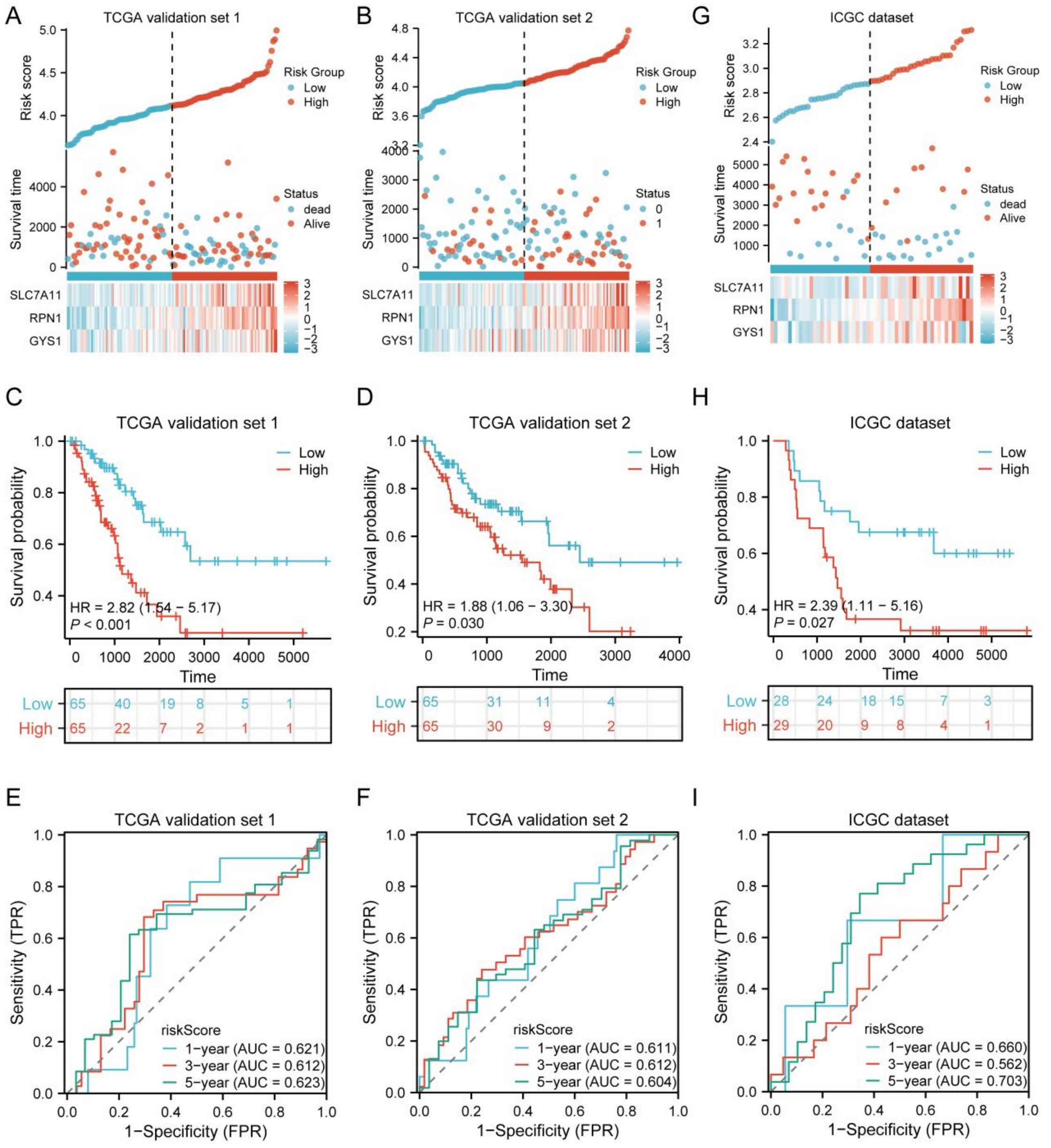

Fig. S3. Prognostic value of DRGs signature and risk score validation in SARC patients. (A) Distribution of risk score, survival status, and expression of prognostic DRGs for patients in low- and high-risk groups in TCGA validation set 1. (B) Distribution of risk score, survival status, and expression of prognostic DRGs for patients in low- and high-risk groups in TCGA validation set 2. (C)Risk score and survival probabilities in TCGA validation set 1. (D) Risk score and survival probabilities in TCGA validation set 2. (E) Time-dependent ROC curve analyses of risk score in TCGA validation set 1.(F) Time-dependent ROC curve analyses of risk score in TCGA validation set 2. (G) Distribution of risk score, survival status, and expression of prognostic DRGs for SARC patients in ICGC cohort; (H) OS curve of SARC patients in high/low-risk groups in ICGC cohort; (I) Time-dependent ROC curve for 1-, 3-, and 5-year OS for DRGs in ICGC cohort.

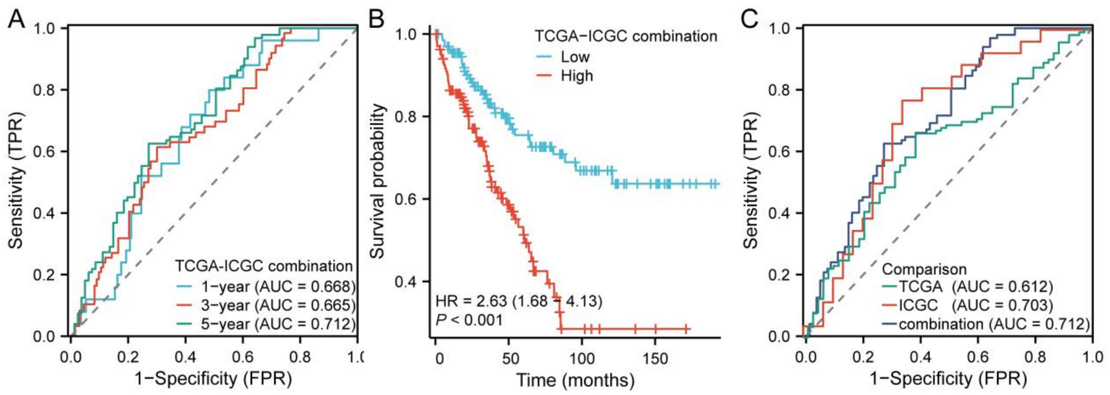

Fig. S4. Comparison and combination of prognostic models. (A) ROC curve analysis combination model predicted the sensitivity and specificity of patients with 1-, 3-, and 5-year OS; (B)Kaplan-Meier survival curves of high-risk and low-risk groups in combination model; (C) ROC curve analyzed the sensitivity and specificity of TCGA, ICGC, and combination models in predicting 5-year OS in patients.

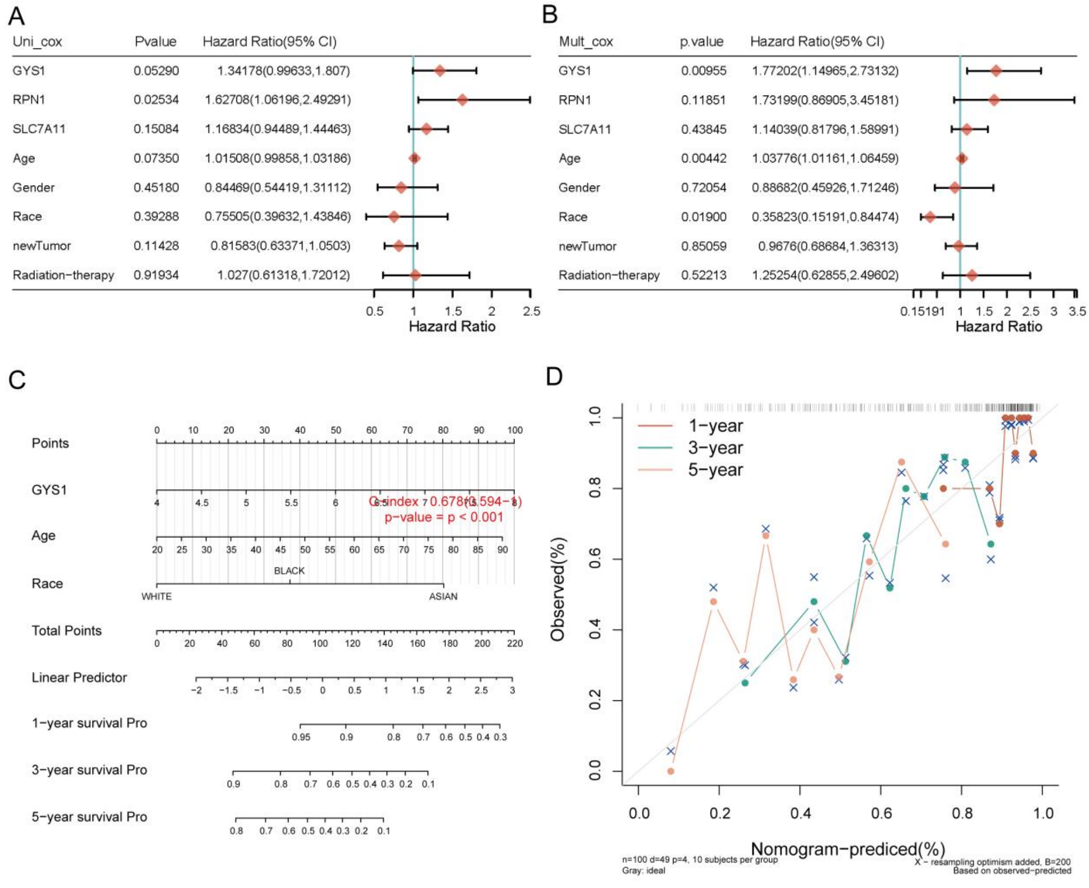

Fig. S5. Construction of a predictive nomogram (DSS). (A, B)Univariate and multivariate Cox regression analysis of clinicopathological features and DRGs in SARC patients for DSS; (C, D) Nomogram for predicting 1-, 3-, and 5-year DSS of SARC patients.

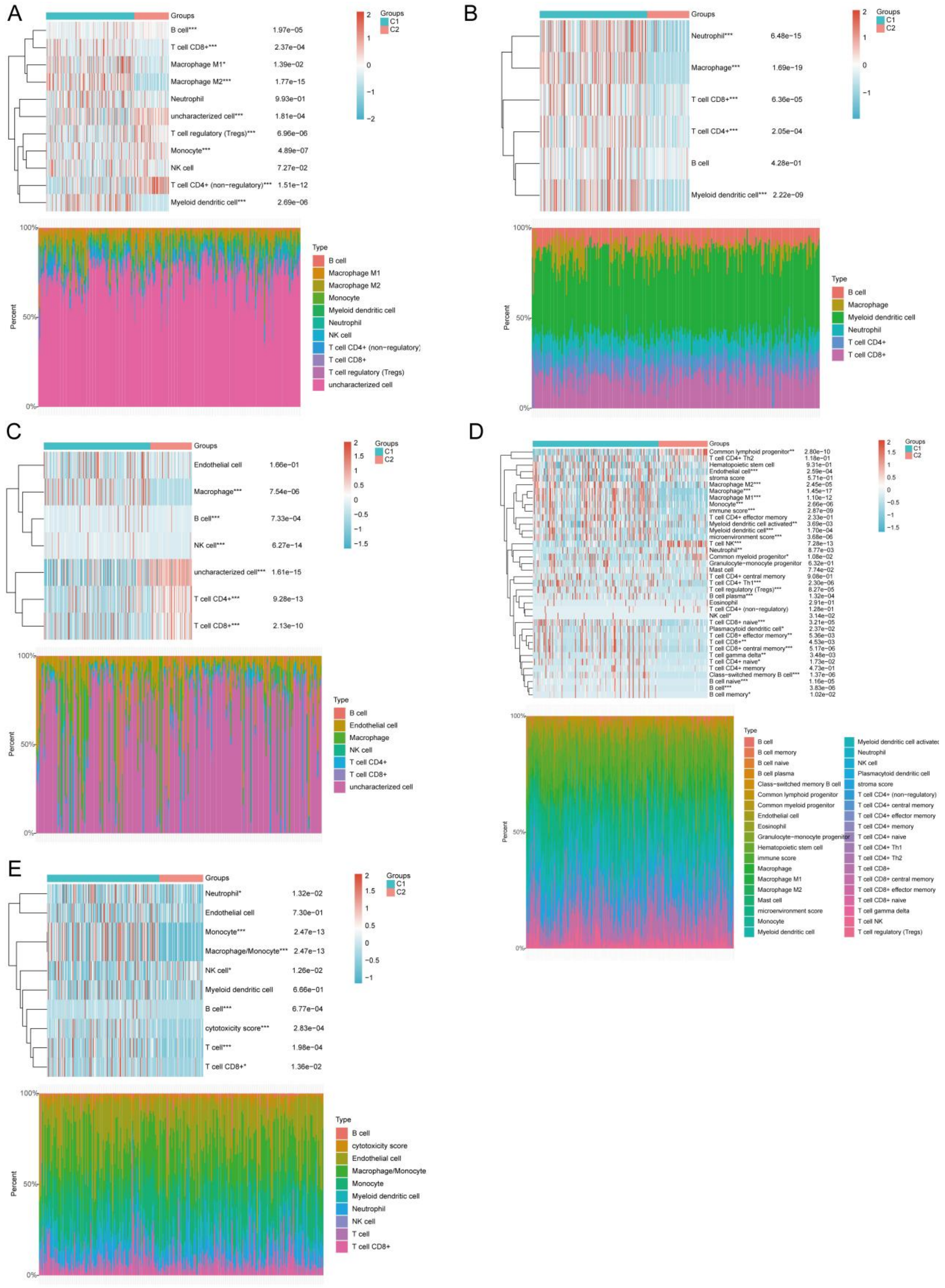

Fig. S6. Relationship between the expression level of DRGs in the tumor microenvironment and immune infiltration (A-E) Comparison of immune scores between C1 and C2 subtypes in TCGA; (A) QUANTISEQ, (B) TIMER, (C) EPIC, (D) xCell, (E) MCPcounter.

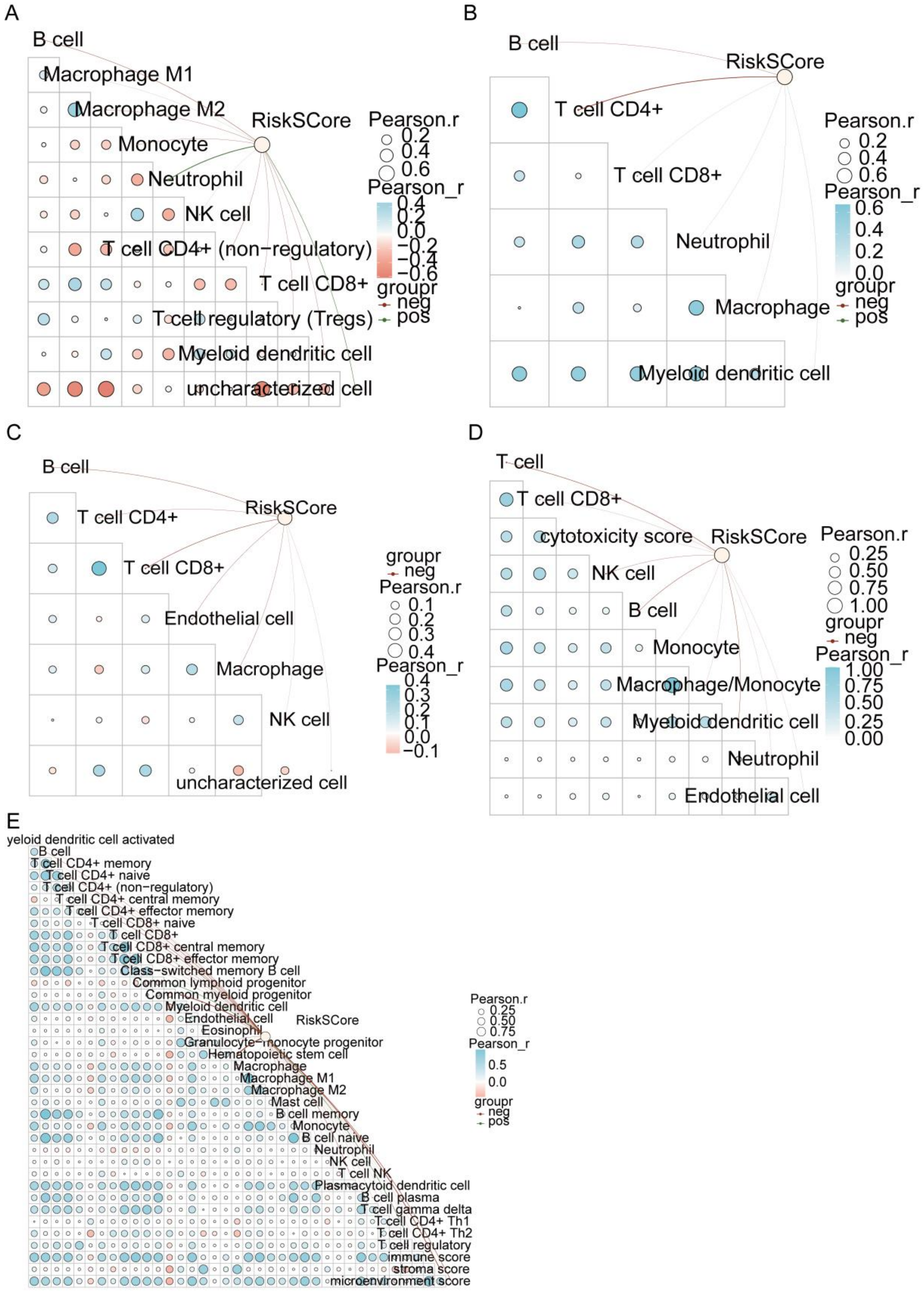

Fig. S7. Relationship between Riskscore and immune scores through different algorithms. (A) QUANTISEQ, (B) TIMER, (C) EPIC, (D) xCell, (E) MCPcounter.

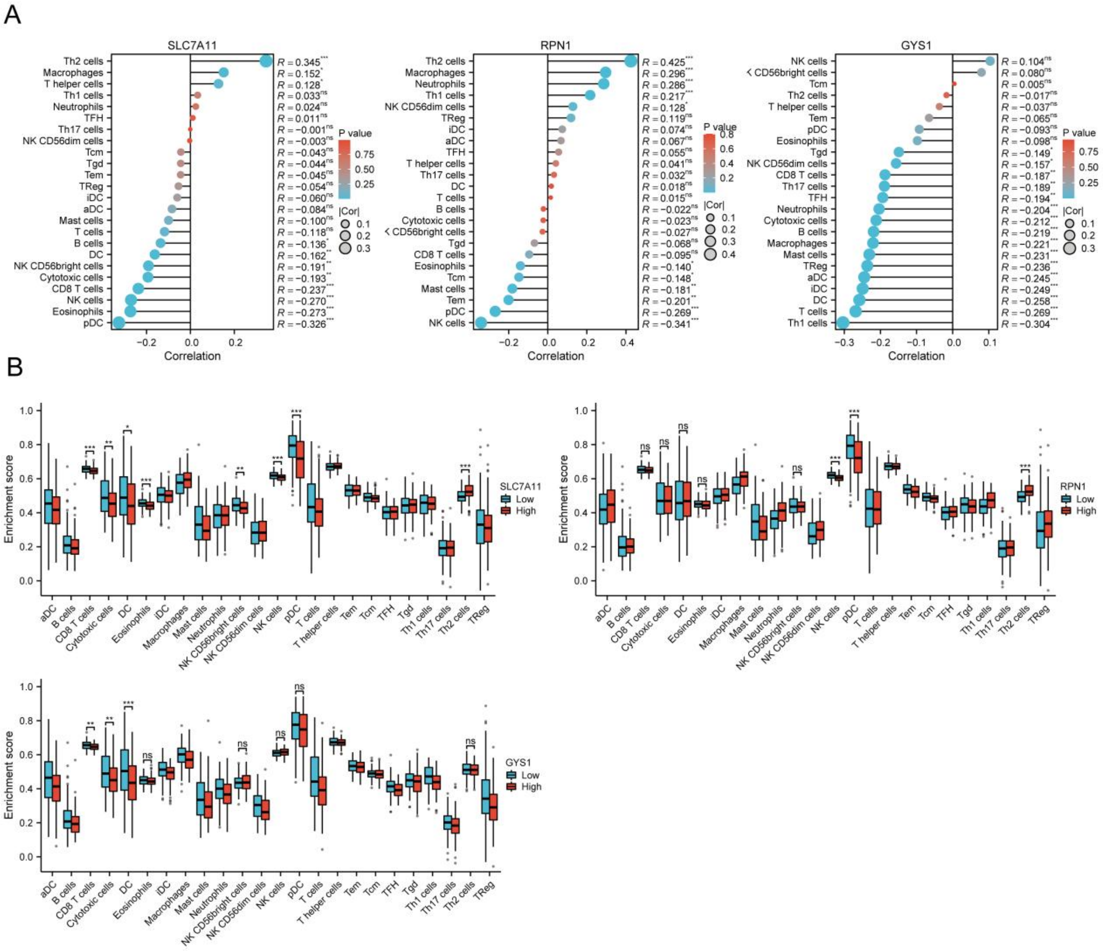

Fig. S8. Relationship between DRGs expression and immune infiltration in SARC. (A) Differences in immune cell infiltration between the high and low expression groups of the three prognostic DRGs in SARC. (B) Correlation between the three prognostic DRGs in SARC and tumor immune cell infiltration.

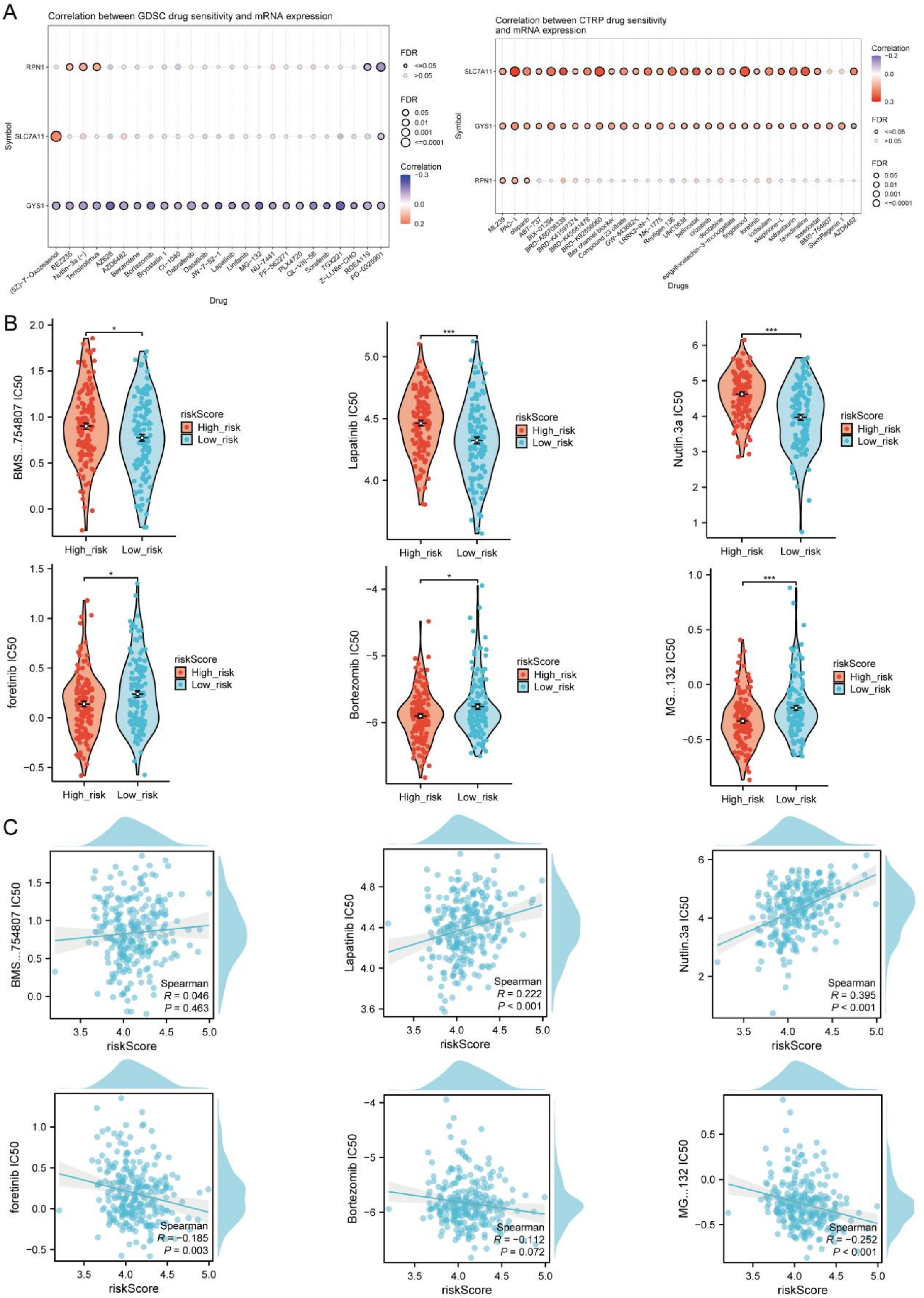

Fig. S9. Drug sensitivity analysis. (A) Predictive antitumor drugs based on the three prognostic DRGs expression in SARC from the GDSC and CTRP datasets. (B)The distribution of IC50 scores in the high and low risk groups; (C)Spearson correlation analysis of IC50 score and riskscore.

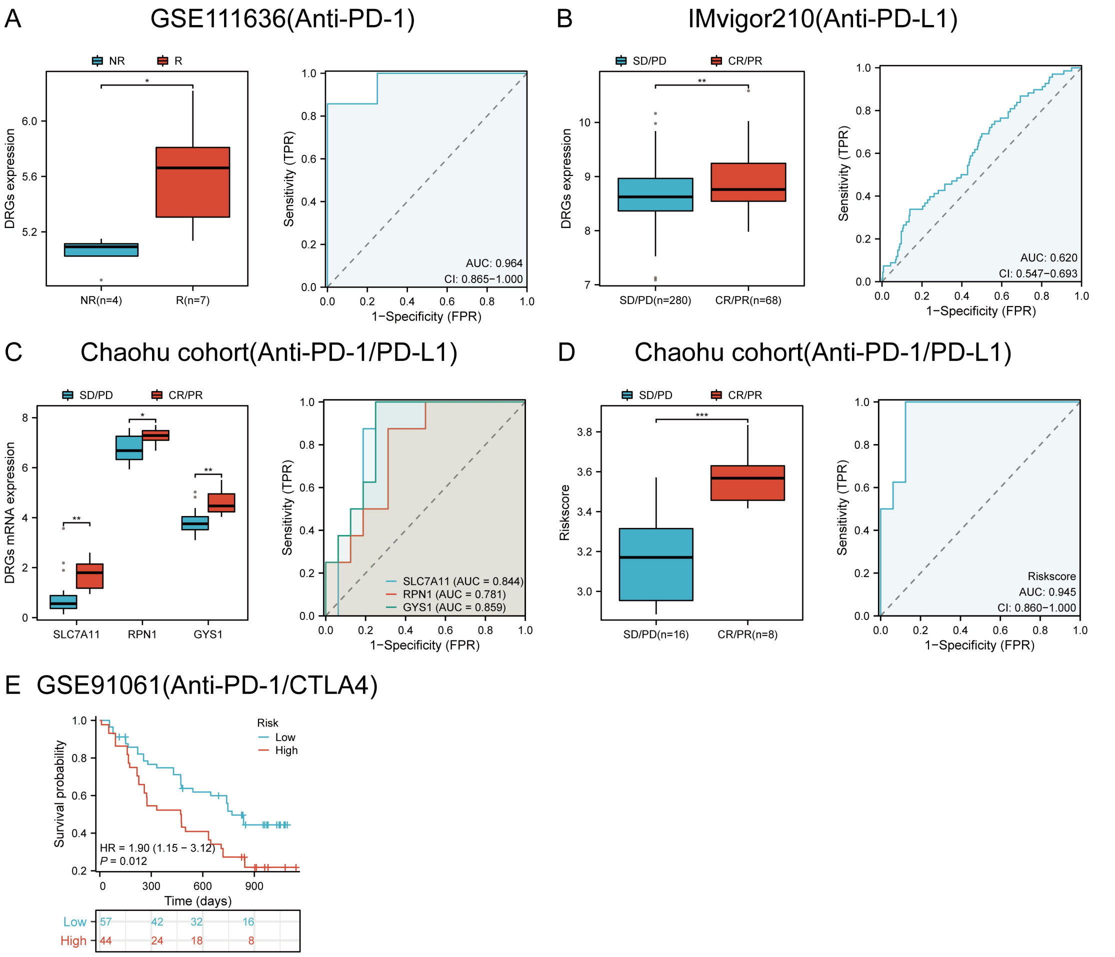

Fig. S10. Immunotherapy response analysis (A)Riskscore differences between patients with NR and R in GSE111636 dataset; ROC analysis of riskscore for prediction of ICI responsiveness in GSE111636 dataset; (B) Riskscore differences between patients with SD/PD and CR/PR in IMvigor210 cohort; ROC analysis of riskscore for prediction of ICI responsiveness in IMvigor210 cohort; (C)Three prognostic DRGs expression differences between patients with SD/PD and CR/PR in Chaohu cohort, respectively; ROC analysis of three prognostic DRGs for prediction of ICI responsiveness in Chaohu cohort, respectively; (D) Riskscore differences between patients with SD/PD and CR/PR in Chaohu cohort; ROC analysis of riskscore for prediction of ICI responsiveness in Chaohu cohort; (E) Kaplan–Meier plots of overall survival for high and low risk patients in GSE91061 dataset. (NR: not responding to immunotherapy. R: respond to immunotherapy. SD/PD: stable disease/progressive disease, CR/PR: complete response/partial response).

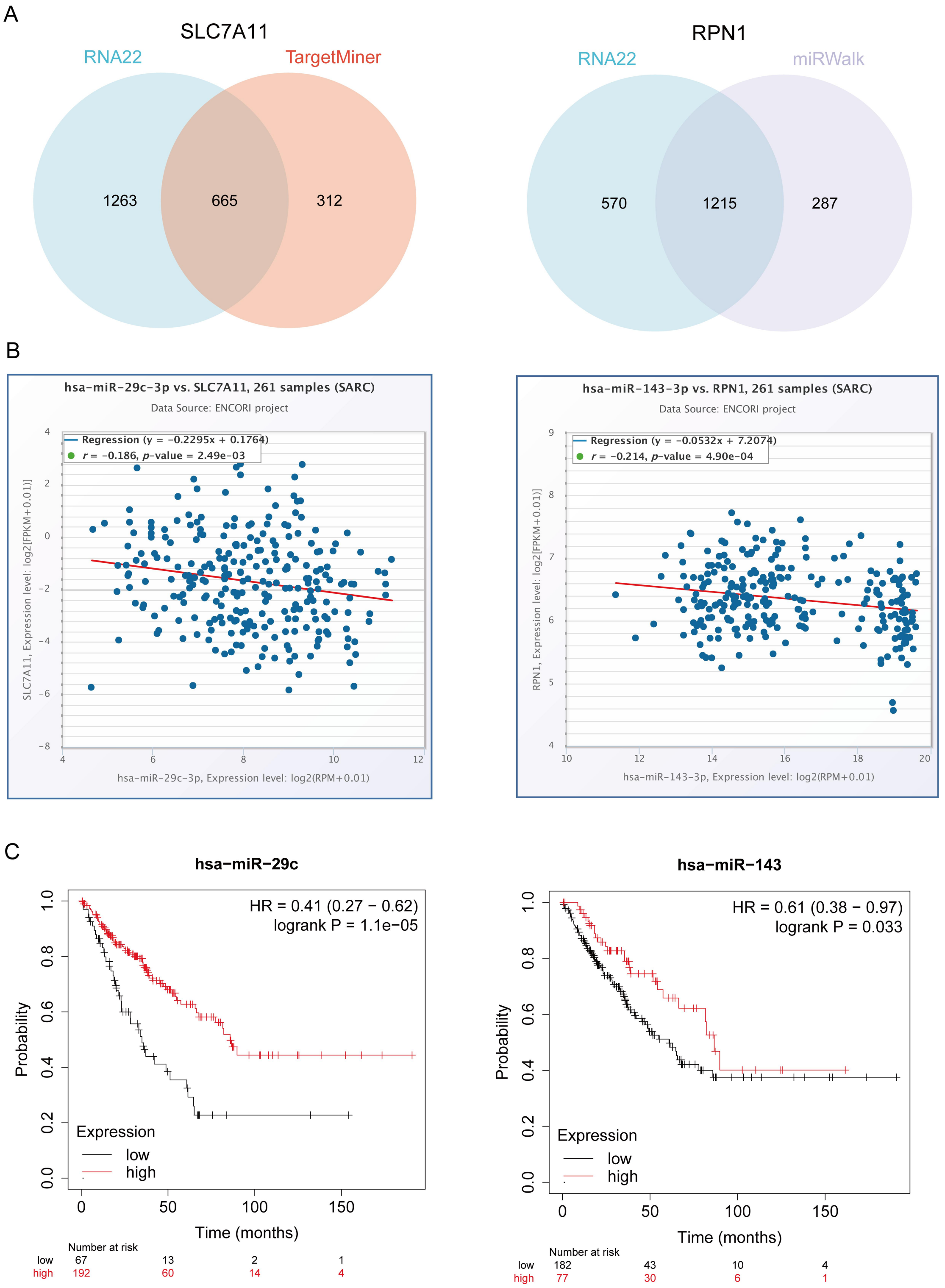

Fig. S11. Identification of the most potential miRNAs associated with the prognosis of SARC. (A) Prediction of the potential miRNAs of the three prognostic DRGs through RNA22, TargetMiner, and miRWalk databases; (B)The correlation between potential miRNAs (hsa-miR-29c-3p, hsa-miR-143-3p) and prognostic DRGs (SLC7A11, RPN1) in SARC; (C) The prognostic value of miRNAs (hsa-miR-29c-3p, hsa-miR-143-3p).

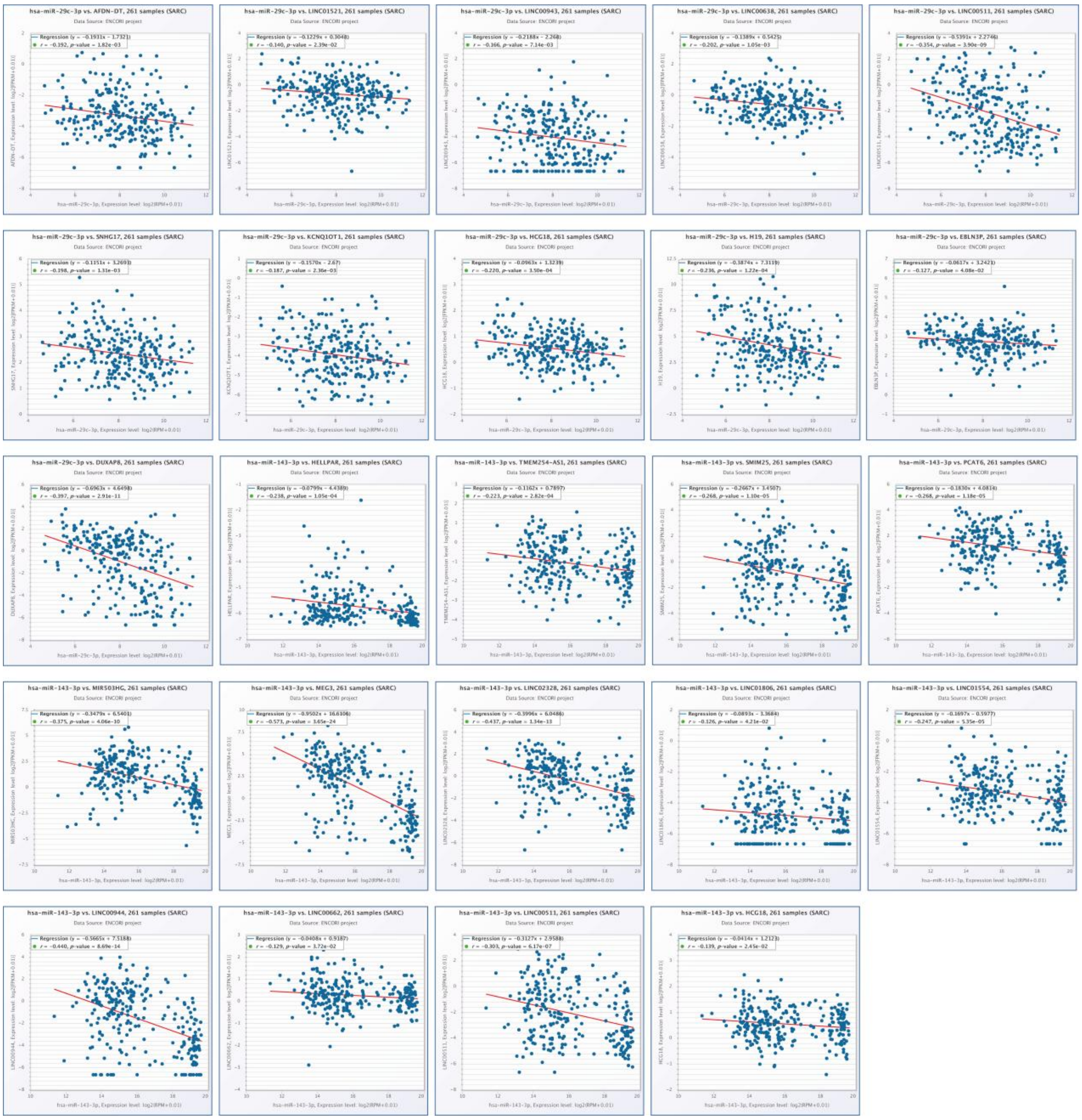

Fig. S12. Correlation of hsa-miR-29c-3p, hsa-miR-143-3p with potential lncRNA target.

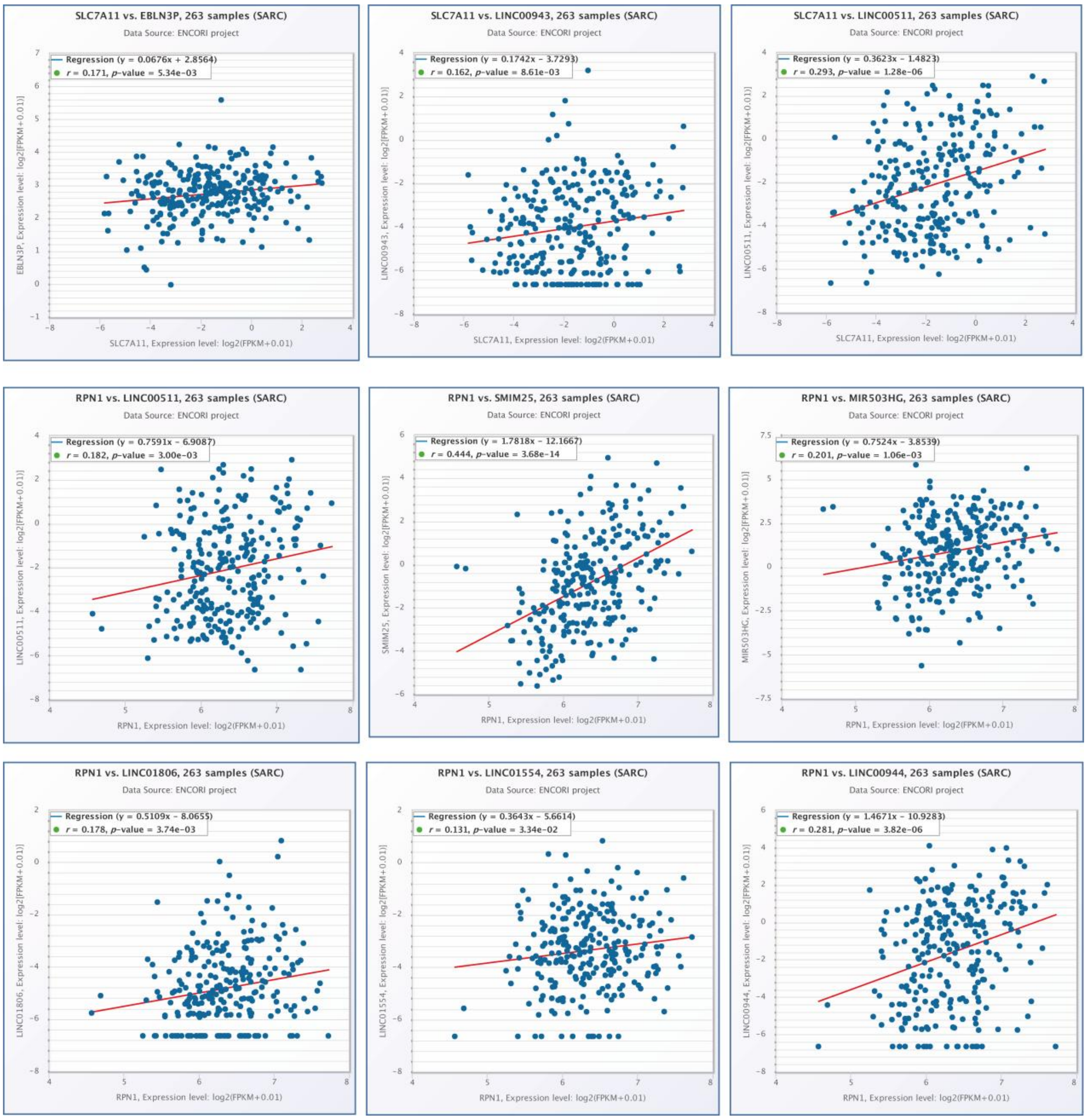

Fig. S13. Correlation of prognostic DRGs with potential lncRNA targets.
